# UPF2-Dependent Nonsense-Mediated mRNA Decay Pathway Is Essential for Spermatogenesis by Selectively Eliminating Longer 3'UTR Transcripts

**DOI:** 10.1371/journal.pgen.1005863

**Published:** 2016-05-05

**Authors:** Jianqiang Bao, Kristoffer Vitting-Seerup, Johannes Waage, Chong Tang, Ying Ge, Bo T. Porse, Wei Yan

**Affiliations:** 1 Department of Physiology and Cell Biology, University of Nevada School of Medicine, Reno, Nevada, United States of America; 2 Biotech Research and Innovation Center (BRIC), University of Copenhagen, Copenhagen, Denmark; 3 The Bioinformatic Centre, Department of Biology, Faculty of Natural Sciences, University of Copenhagen, Copenhagen, Denmark; 4 The Finsen Laboratory, Rigshospitalet, Faculty of Health Sciences, University of Copenhagen, Copenhagen, Denmark; 5 Danish Stem Cell Centre (DanStem), Faculty of Health Sciences, University of Copenhagen, Copenhagen, Denmark; University of Basel, SWITZERLAND

## Abstract

During transcription, most eukaryotic genes generate multiple alternative cleavage and polyadenylation (APA) sites, leading to the production of transcript isoforms with variable lengths in the 3’ untranslated region (3’UTR). In contrast to somatic cells, male germ cells, especially pachytene spermatocytes and round spermatids, express a distinct reservoir of mRNAs with shorter 3’UTRs that are essential for spermatogenesis and male fertility. However, the mechanisms underlying the enrichment of shorter 3’UTR transcripts in the developing male germ cells remain unknown. Here, we report that UPF2-mediated nonsense-mediated mRNA decay (NMD) plays an essential role in male germ cells by eliminating ubiquitous genes-derived, longer 3’UTR transcripts, and that this role is independent of its canonical role in degrading “premature termination codon” (PTC)-containing transcripts in somatic cell lineages. This report provides physiological evidence supporting a noncanonical role of the NMD pathway in achieving global 3’UTR shortening in the male germ cells during spermatogenesis.

## Author Summary

3’UTR length control has been identified as a critical mechanism through which the cell establishes and maintains its functional identity. Developing male germ cells, especially spermatocytes and spermatids, display a transcriptome enriched in short 3’UTR transcripts, which has been demonstrated to be essential for spermatogenesis. However, it remains unknown how global 3’UTR shortening is achieved. Here, we report that most of the genes, especially those ubiquitously expressed, are transcribed into multiple isoforms in spermatocytes, and when spermatocytes develop into round spermatids, those long 3’UTR transcripts are selectively degraded by UPF2-dependent nonsense-mediated mRNA decay (NMD), leading to enrichment of the shorter 3’UTR transcripts. We provide physiological evidence supporting a non-canonical role of NMD in the control of 3’UTR length in male germ cells.

## Introduction

Spermatogenesis is a complex cellular differentiation process through which male germline stem cells develop sequentially into spermatogonia, spermatocytes, spermatids, and eventually spermatozoa [1]. Both meiosis (i.e. spermatocyte development) and spermiogenesis (i.e. spermatid differentiation into spermatozoa) are unique to male germ cell development. In both processes, a large number of protein-coding genes are transcribed without immediate translation, a phenomenon that has been termed “uncoupling of transcription and translation” [2, 3]. The delayed translation results from the cessation of transcription in step 9 spermatids due to the onset of chromatin condensation and elongation. For example, mRNAs for protamines (*Prm1 and Prm2*) and transition proteins (*Tnp1 and Tnp2*) are transcribed in late pachytene spermatocytes, but are not translated until ~one week later when spermatocytes have developed into elongating spermatids in mice [4, 5]. These mRNA transcripts are sequestered in ribonucleoprotein particles (RNPs), in which the mRNA transcripts are stabilized by RNA-binding proteins (RBPs) and small noncoding RNAs (e.g., miRNAs), and physically separated from the translational machinery. In elongating and elongated spermatids, these transcripts can translocate and get loaded onto the polyribosomes for translation when specific proteins are needed for sperm assembly [3, 6]. In addition to delayed translation, the transcriptome of meiotic and haploid male germ cells (i.e., spermatocytes and spermatids) is characterized by the enrichment of mRNA transcripts bearing shorter 3’UTRs, which is not shared by most of somatic cell types [6–9]. Given that transcription and translation are uncoupled, enhanced stability and translational efficiency are critical for accurate spatiotemporal expression of a large number of proteins required for sperm assembly during late spermiogenesis [3]. Transcripts with shorter 3’UTRs have been shown to be more stable and more efficient in translation due to the reduced binding sites for RBPs and miRNAs [10, 11], which may explain why a repertoire of shorter 3’UTR mRNAs is exclusively expressed during meiosis and spermiogenesis and they are essential for both processes [6–9].

Processing of the 3’ ends of mRNA transcripts is necessary for mRNA maturation and involves the cleavage at the polyadenylation site (PAS) by a nuclear endonuclease followed by the addition of a stretch of adenosines (PolyA tail). Notably, the usage of alternative PAS sites and polyadenylation, termed as alternative cleavage and polyadenylation (APA), is a common event in eukaryotic gene transcription, which leads to the generation of mRNA transcripts with variable 3’UTR lengths. In general, the upstream and downstream sequences flanking the PAS cleavage site in a pre-mRNA serve as the cis-elements, which are specifically recognized and bound by the core APA factors. The APA machinery consists of cleavage and polyadenylation specificity factor (CPSF) proteins, the cleavage stimulation factor (CstF) proteins, and cleavage factor I. Together with auxiliary and tissue-specific protein factors (e.g., *Nova1* in neuron) [12], the APA complex generates temporal or tissue-specific mRNA transcriptomes enriched for mRNAs with different 3’UTR lengths. For example, recent high-throughput sequencing studies have identified that mRNAs with the longest 3’UTRs are predominately present in brain, whereas the testis tends to be enriched in mRNA isoforms with shorter 3’UTRs [13, 14]. Interestingly, the differential usage of alternative PAS sites is widely observed under stress conditions [15], in proliferating/cancer cells [16, 17], through early embryonic development [18], and during induced somatic cell reprogramming [19].

Although the enrichment of shorter 3’UTR transcripts in the testis has been known for decades [20], the underlying mechanism remains elusive [8]. The current dogma emphasizes the biased production of testis-specific transcripts with shorter 3’UTRs through testis-specific APA factors, which prefer the proximal to distal polyadenylation sites, thus achieving global 3’UTR shortening in the testis [6, 8]. However, such factors remain yet-to-be-identified.

Alternative splicing (AS) is a common form of post-transcriptional regulation observed in ~75%-90% of human protein-coding genes whereby one gene generates multiple isoforms of mRNA transcripts with variable stability and translational efficiency as well as distinct protein-coding potential [21]. Concomitantly, it has been estimated that one third of the AS events also create aberrant transcript isoforms that would trigger nonsense-mediated mRNA decay (NMD) [22]. The NMD pathway is highly conserved across all eukaryotes, and serves as a critical cellular surveillance mechanism by eliminating aberrant mRNA transcripts harboring the so-called “premature termination codon” (PTC), which generally resides >50nt upstream of the last exon-exon junction (i.e., “the 50nt rule”) [23–25]. In mammalian somatic cells, the core NMD machinery includes three trans-acting factors: UPF1, UPF2 and UPF3, in addition to SMG1-7 [23, 24]. UPF2 is considered as a molecular linker that bridges the interaction between UPF3, which is bound to the exon-exon junction complex (EJC), and UPF1-containing complex (SURF) recruited to the stalled ribosome, constituting the core NMD complex that subsequently stimulates phosphorylation of UPF1 to induce decay activity [26]. Supporting its well-established role in eliminating PTC-containing mRNA transcripts during translation [23, 24], earlier *in vitro* studies using cell lines deficient in NMD activity have reported a conspicuous upregulation of a substantial proportion (up to 60%) of PTC-positive mRNA transcripts [27–30]. Our *in vivo* study using conditional *Upf2* knockout mice also demonstrates a global upregulation of ~one third of PTC-positive transcripts in liver and bone marrow [31].

Classical NMD substrates include those transcripts bearing PTC that resides >50 nucleotide upstream of the final exon-exon junction complex (EJC) [25]. During translation, the ribosomes stall in the PTC, resulting in the failure to remove the downstream EJC complex, which, in turn, promotes NMD-mediated degradation of these PTC-positive transcripts [24, 26]. In addition to the classical EJC-dependent NMD, more recent genome-wide studies identified that NMD not only degrades mRNA substrates harboring PTCs, but also regulates a selection of normal mRNA transcripts encoding full-length proteins devoid of PTCs through an EJC-independent NMD mechanism [27, 31–33]. These studies significantly expand the scope of NMD target repertoire, and strongly suggest a critical, physiological role of the NMD pathway in regulating the transcriptomic homeostasis in addition to its canonical roles in eliminating the PTC-positive transcripts [27, 32, 33]. One such EJC-independent, NMD-triggering feature identified is the 3’UTR length. *In vitro* cell lines-based studies have demonstrated that transcripts with aberrant 3’UTR architecture are more susceptible to NMD [32, 34, 35]. However, physiological evidence from loss- or gain-of-function studies *in vivo* to support this notion still remains missing.

We were intrigued to explore whether the NMD pathway plays an essential role in male germ cells by inactivating UPF2, a core NMD factor, specifically in the male germline. Surprisingly, we observed a weak, canonical role of the NMD pathway in degrading the PTC-positive transcripts, but a significant, noncanonical role in selective degradation of mRNA isoforms bearing longer 3’UTRs that are often derived from ubiquitously expressed genes. Our data provide physiological evidence supporting that the 3’UTR-shortened, testis-specific transcriptome is established through, at least in part, eliminating longer 3’UTR transcripts derived from ubiquitously expressed genes by the UPF2-mediated NMD.

## Results

### UPF2 is a novel component of the chromatoid body (CB)

**Fig 1:**
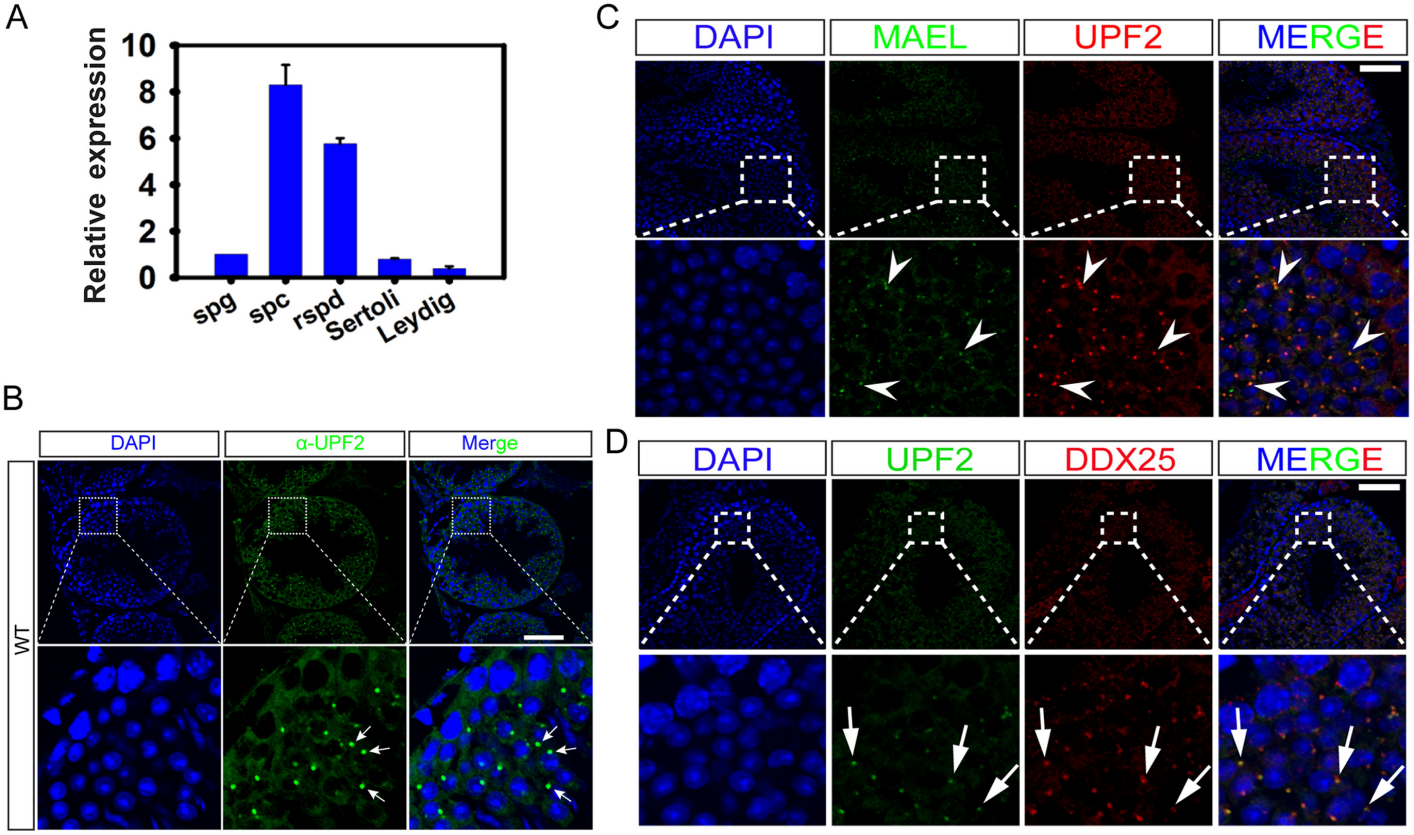
UPF2 is a novel component of the chromatoid body (CB) and is highly expressed in spermatocytes and round spermatids in murine testes. (A) qPCR analyses of *Upf2* mRNA levels in individual testicular cell types purified from adult murine testes, including spermatogonia (spg), spermatocytes (spc), round spermatids (rspd), Sertoli cells (Sertoli) and Leydig cells (Leydig). Biological triplicates (n = 3) were analyzed and relative *Upf2* mRNA levels are shown as means ± SEM. (B) Immunofluorescent localization of UPF2 in WT murine testes. UPF2 is abundantly expressed in the cytoplasm of spermatocytes and round spermatids in WT testes with the highest expression confined to an intensive “dot-like” structure, resembling the chromatoid body (CB, arrows), in round spermatids. Scale bar = 50μm. (C) Double immunofluorescent staining of UPF2 and MAEL, a CB marker, in adult murine testes. Arrowheads indicate CBs in round spermatids. Scale bar = 50μm. (D) Co-localization of UPF2 and DDX25, a CB marker protein. In both stages VIII and I seminiferous tubules, UPF2 mostly overlaps with DDX25 in CBs in the round spermatids (arrows). However, UPF2 is absent in a few DDX25-positive dots (arrowheads), which may represent other types of cytoplasmic granular structures, e.g. the satellite body. Scale bar = 15μm.

To study the testicular function of UPF2, we first examined its expression and localization in developing and adult testes. Among multiple adult organs examined, UPF2 protein was preferentially expressed in testes (S1 Fig). *Upf2* mRNAs became detectable in fetal testes and the levels increased gradually with the postnatal testicular development (S1B Fig). In adult testes, mRNAs for *Upf2* and other seven well-known nonsense-mediated decay (NMD) factors, including *Upf1*, *Upf3a*, *Upf3b*, *Smg1*, *Smg5*, *Smg6* and *Smg7*, were all predominantly detected in spermatocytes and spermatids (Figs 1A and S1). Immunofluorescent staining with a well-characterized UPF2 antibody [36] revealed that UPF2 protein was mainly localized to the cytoplasm of spermatocytes and spermatids (Fig 1B). Interestingly, UPF2 became highly concentrated to a perinuclear structure resembling the “chromatoid body” (CB) in round spermatids (Fig 1B). The CB is a highly conserved, cloud-like perinuclear structure that moves around the nuclear pores in the cytoplasm of round spermatids, and has been suggested to serve as a RNA processing center essential for spermatogenesis [37, 38]. To further explore if UPF2 is a CB component, we performed co-immunostaining for both UPF2 and MAEL, a CB marker [39] on adult testicular cryosections. The majority (>90%) of the UPF2-positive “dots” co-localized with the MAEL-positive foci in round spermatids (Fig 1C). Consistently, most (>90%) of the UPF2-positive “dots” also overlapped with the signals of DDX25, another well-characterized CB marker, in round spermatids (Fig 1D). Together, these data suggest that UPF2, as a novel integral component of the CB, may play an important role in male germ cells, especially in spermatocytes and spermatids, by regulating RNA processing.

### Selective inactivation of *Upf2* in prospermatogonia leads to “Sertoli-only syndrome”

**Fig 2:**
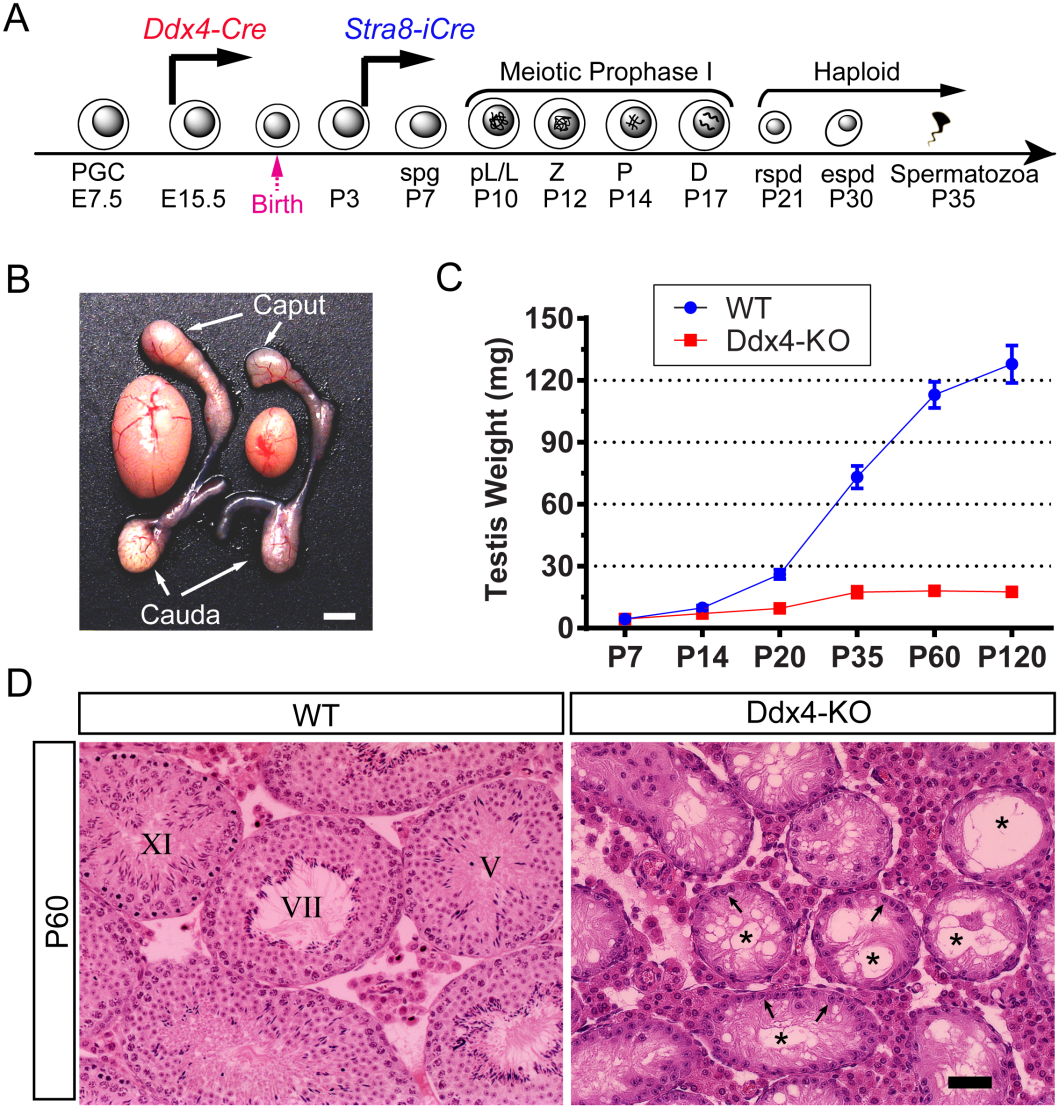
UPF2 is essential for prospermatogonial development in murine testes. (A) A schematic diagram showing the critical time points during male germ cell development. Two Cre deletor lines used express Cre in the male germline starting from E15.5 (*Ddx4-Cre*) and P4 (*Stra8-Cre*) as indicated. PGC, primordial germ cell; E, embryonic day; spg, spermatogonium; pL, pre-leptotene spermatocyte; L, leptotene spermatocyte; Z, zygotene spermatocyte; P, pachytene spermatocyte; D, diplotene spermatocyte; rspd, round spermatid; espd, elongating/elongated spermatid. (B) Gross morphology of the testis and the epididymis of WT and Ddx4-KO mice. (C) Testis growth curves of WT and Ddx4-KO mice. Testis weight is presented as means ± SEM (n = 6). (D) Testicular histology of WT and Ddx4-KO mice at P60. Seminiferous tubules of Ddx4-KO mice were devoid of germ cells and suffused with vacuoles (*). Only Sertoli cells (arrows) were present along the basal membrane. Scale bar = 30μm.

**Fig 3:**
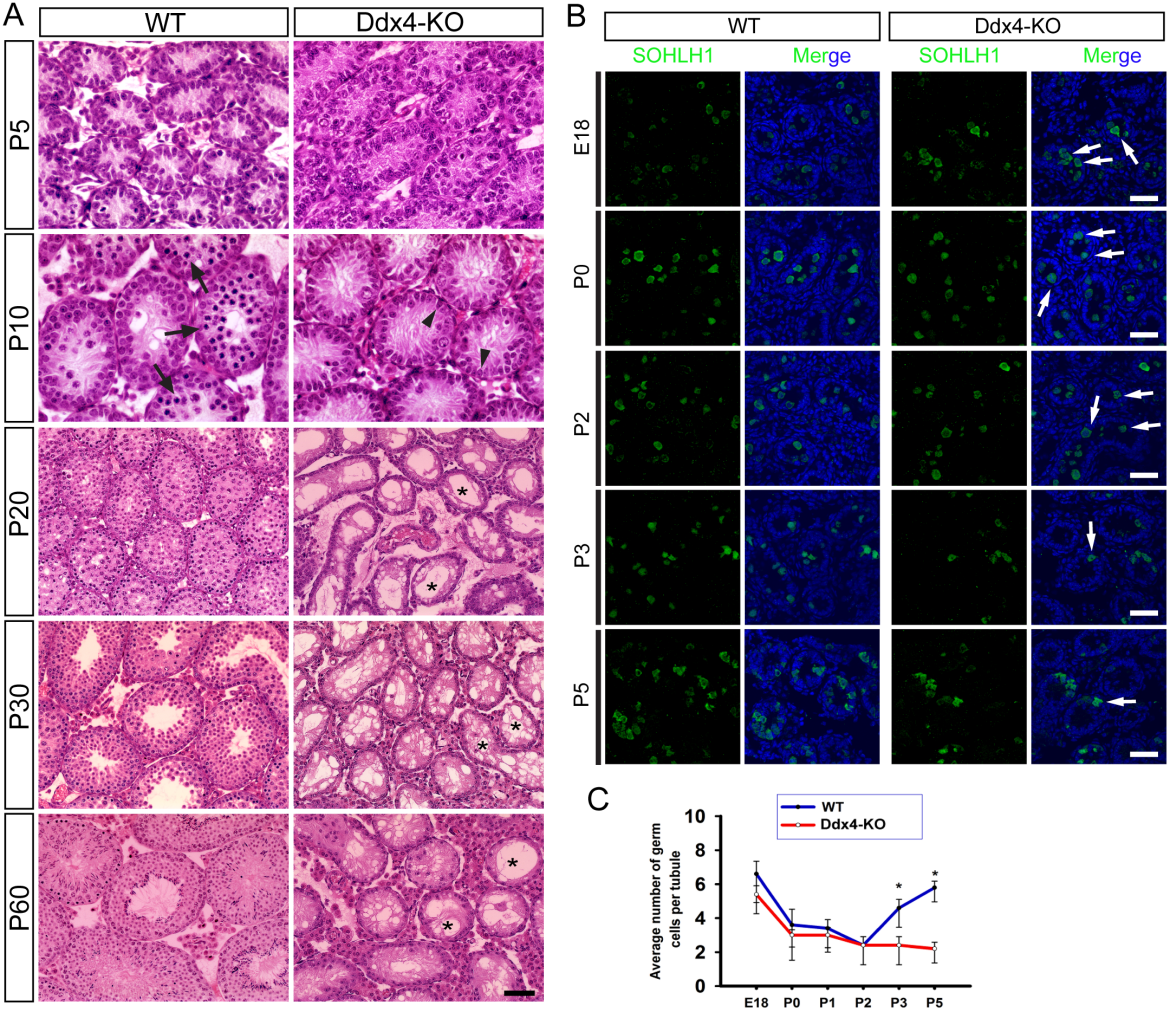
Histological analyses of the developing testes in WT and Ddx4-KO mice. (A) HE staining of paraffin-embedded testicular sections. At postnatal day 5 (P5), the number and morphology of germ cells and Sertoli cells are indistinguishable between WT and Ddx4-KO testes. Upon P10, preleptotene or leptotene spermatocytes (arrows) started to appear in WT testes, but not in Ddx4-KO testes due to germ cell loss and delay in meiotic entry. At P20 and thereafter, numerous vacuoles (*) were present, indicative of massive germ cell depletion in Ddx4-KO testes. Scale bar = 50μm. (B) Loss of prospermatogonia during neonatal testicular development in Ddx4-KO males. Immunostaining of SOHLH1, a marker for prospermatogonia and spermatogonia, in the testes of Ddx4-KO male mice at embryonic day 18 (E18), postnatal day 0 (P0), P2, P3 and P5. Scale bar = 70μm. (C) Quantitative analyses of the average number of germ cells per tubule cross-section in WT and Ddx4-KO testes at E18, P0, P2, P3 and P5. Data are presented as Means ± SD, n = 20. * denotes statistical significance (P<0.05).

To define the physiological role of UPF2 in male germline development, we first generated prospermatogonia-specific *Upf2* conditional knockout mice (*Ddx4-Cre; Upf2*^*fl/Δ*^, hereafter called Ddx4-KO) by crossing *Ddx4-Cre* [40] with *Upf2* floxed (*Upf2*^*fl/fl*^) mice [41] (Fig 2A). The Cre activity first becomes detectable exclusively in primordial germ cells on embryonic day 15.5 (E15.5) in *Ddx4-Cre* mice [40] and thus, the floxed *Upf2* allele is expected to be deleted in prospermatogonia and all subsequent male germ cell types (S2 Fig). All adult Ddx4-KO males were infertile and exhibited a drastic reduction in testis size compared to age-matched WT controls (Fig 2B). Marked testicular atrophy was observed during postnatal development in Ddx4-KO males (Fig 2C). Consistently, histological examination revealed that adult Ddx4-KO seminiferous tubules contained no or few spermatogenic cells, but numerous vacuoles, indicative of massive germ cell depletion (Fig 2D). Discernable histological differences between Ddx4-KO and WT testes were observed at as early as postnatal day 10 (P10) (Fig 3A). However, the Ddx4-KO males already displayed a reduced total number of germ cells at P3 (Fig 3B and 3C), as revealed by immunostaining using a germ cell-specific protein marker SOHLH1 [42, 43]. By P10, co-immunostaining for both WT1 (a Sertoli cell-specific marker) and GCNA (a germ cell-specific marker) [44] revealed that very few germ cells remained in the Ddx4-KO seminiferous tubules (S3 Fig), indicating that *Upf2*-null prospermatogonia/spermatogonia were rapidly depleted during neonatal testicular development in Ddx4-KO testes. Seminiferous tubules in adult Ddx4-KO testes contained mostly Sertoli cells, resembling the “Sertoli-only syndrome” in men [45]. Taken together, these data demonstrate that *Upf2* is required for prospermatogonial development.

### Conditional ablation of *Upf2* in postnatal male germ cells causes azoospermia and male sterility

**Fig 4:**
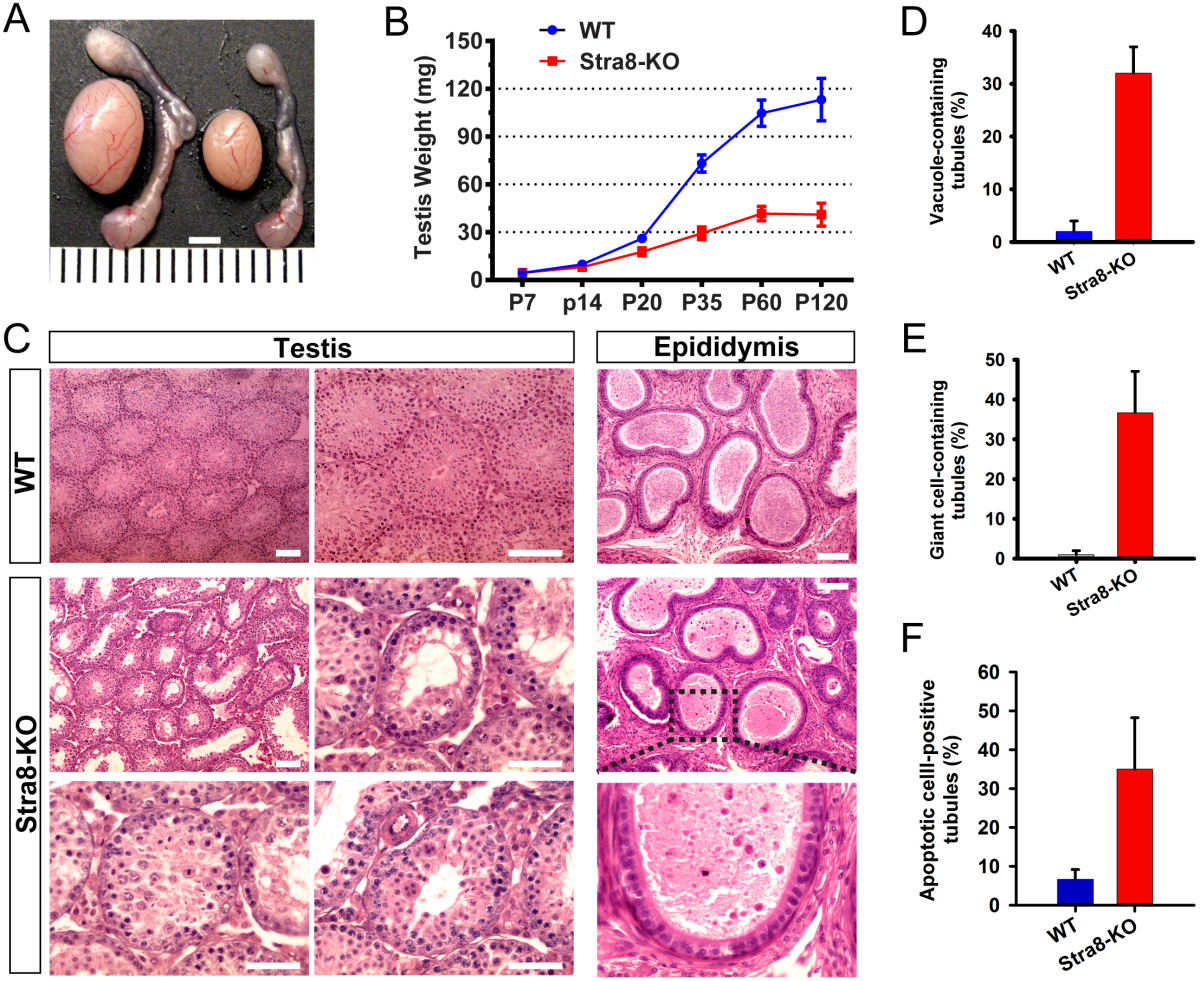
UPF2 is required for postnatal germ cell development in mice. (A) Gross morphology of the testis and the epididymis of WT and Stra8-KO mice. Scale bar = 2mm. (B) Testis growth curve during postnatal development between WT and Stra8-KO mice (n = 6 for each genotype). (C) Histology of WT and Stra8-KO testes and epididymides at P60. While abundant mature spermatozoa were observed in the WT cauda epididymis, only a few degenerating, immature male germ cells were present in the Stra8-KO cauda epididymis. (D-E) Percentage of seminiferous tubules containing vacuoles (D) or multinucleated giant cells (E) in WT and Stra8-KO testes (n = 4 for each genotype). (F) Percentage of tubules containing apoptotic cells based on terminal deoxynucleotidyl transferase dUTP nick end labeling (TUNEL) assay; Scale bar = 50μm.

Predominant expression of UPF2 in spermatocytes and round spermatids in adult testes implicates a critical role of UPF2 in the meiotic and haploid phases of spermatogenesis. To define this role, we generated the *Stra8-Cre; Upf2*^*fl/Δ*^ (hereafter called Stra8-KO) mice line, in which *Upf2* was specifically deleted in meiotic and haploid male germ cells [46, 47] (Figs 2A and S2). All adult Stra8-KO males were infertile and exhibited a significant reduction (~60%) in testis weight compared to WT controls (Fig 4A and 4B). Histological analyses revealed that zygotene spermatocytes were present in both WT and Stra8-KO seminiferous tubules at P12. Starting from P14, multiple defects, including delayed meiotic entry and massive depletion of spermatocytes and spermatids, were observed in Stra8-KO developing testes (S4 Fig). In adult Stra8-KO testes, numerous vacuoles and multinucleated giant cells were present in the seminiferous epithelia (Figs 4C–4F and S4), indicative of massive depletion of spermatocytes and round spermatids. Consequently, no sperm were present in the cauda epididymis in Stra8-KO males (Fig 4C). These data suggest that *Upf2* is essential for not only the first wave of spermatogenesis during testicular development, but also the subsequent spermatogenic cycles in adult testes.

### No global PTC upregulation in *Upf2*-deficient pachytene spermatocytes or round spermatids

**Fig 5:**
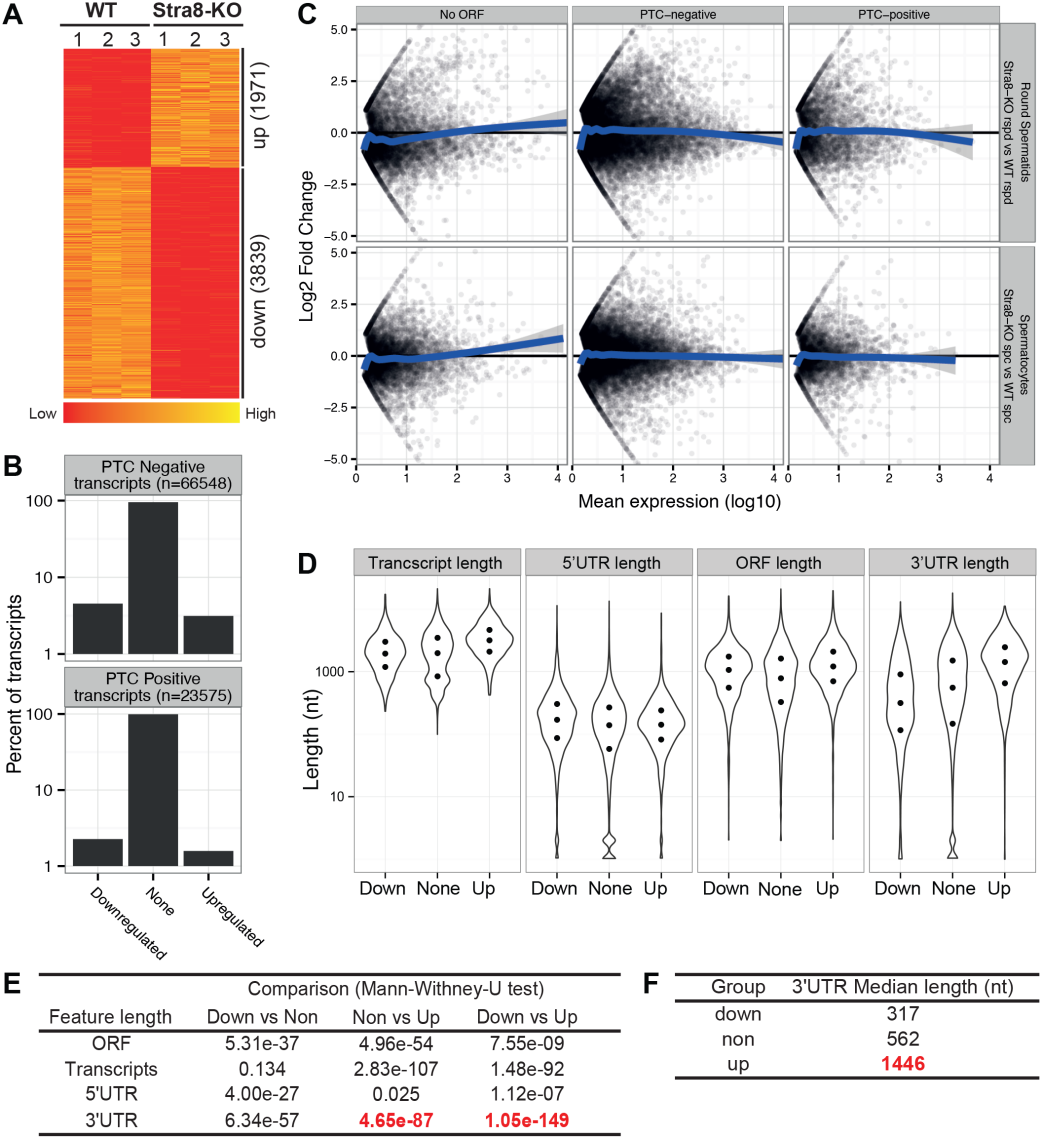
RNA-Seq analyses reveal no global PTC upregulation, but a drastic increase in the 3’UTR length among upregulated, ubiquitously expressed transcripts in Stra8-KO total testes or purified germ cells. (A) Heat map showing the clustering of de-regulated transcripts identified through RNA-Seq analyses in Stra8-KO testes. A total of 3,839 transcripts were significantly downregulated, while 1,971 transcripts were significantly upregulated in Stra8-KO total testes (Cutoff: FDR<0.05). (B) PTC analyses of RNA-Seq data demonstrated that among all 23,575 PTC-positive transcripts (17,6% of all transcripts), only 137 were upregulated (~7% of upregulated), suggesting a lack of global PTC upregulation in Stra8-KO testes. All de-regulated genes and transcripts are listed in S1 Table. (C) MA plots of transcript expression showing no global upregulation of PTC-positive transcripts in either *Upf2*-null spermatocytes (spc) or *Upf2*-null round spermatids (rspd). Transcripts without annotated start codons (NO ORF), transcripts without PTC (PTC-negative), and transcripts containing PTC (PTC-positive) were analyzed. The blue line indicates the mean log2 fold change along the x-axis. (D) Violin plots showing the length distribution of full-length transcript, ORF, 5’UTRs and 3’UTR among up- (1,559), down- (2,654) and non-regulated (85,910) transcripts in Stra8-KO testes (Cutoff: FDR < 0.05). Only transcripts derived from genes expressing multiple transcripts were analyzed. The three dots in each violin plot indicate the 25^th^, 50^th^ (median) and 75^th^ percentile of the visualized data. (E) Statistics for the pairwise comparisons of the length distributions shown in D. The most significant length increases were observed in 3’UTRs of the up-regulated transcripts in Stra8-KO testes (p-value = 1.05e-149, between up- and down- groups). (F) The median 3’UTR length of the up-, down- and non-regulated transcripts analyzed in D.

The well-known canonical function of the NMD machinery is to eliminate PTC-containing transcripts, which are often derived from aberrant alternative splicing of pre-mRNAs [7, 48]. Indeed, we have previously demonstrated that selective inactivation of *Upf2* leads to the upregulation of ~one third of the PTC-positive transcripts in liver and bone marrow [31]. Given the pronounced alternative splicing activities in developing male germ cells, especially in spermatocytes and round spermatids, we hypothesized that *Upf2* inactivation would lead to an accumulation of, alternatively spliced PTC-bearing transcripts, which would be deleterious to spermatogenesis. To test this hypothesis, we performed RNA-Seq analyses using WT and Stra8-KO total testes in biological triplicates at the age of 6 weeks, a time point when the first spermatogenic cycle was fully completed. Full-length transcripts were re-constructed based on the paired-end RNA-Seq data using Cufflinks [49]. The full-length transcripts were then analyzed for PTC using the R package spliceR [50], which annotates transcripts as PTC-positive, if a stop codon is found >50nt upstream of the last exon-exon junction. Surprisingly, we found that of the 1,971 up-regulated transcripts identified in Stra8-KO testes (FDR <0.05), only 137 (~7%) contains a PTC (Fig 5A and 5B). This is far less than the >30% previously found in somatic *Upf2-*null cells [27, 31]. As mentioned earlier, active depletion of *Upf2*-deficient spermatocytes and spermatids were observed during the first wave of spermatogenesis (Figs 3A and S4). To exclude the possibility that the disproportional cell constituents due to germ cell depletion in Stra8-KO total testes may have masked the upregulated PTC-positive transcripts, we further conducted RNA-Seq analyses using spermatocytes and round spermatids purified and pooled from WT and Stra8-KO total testes (see methods). qPCR analyses further confirmed the absence of *Upf2* mRNAs in *Upf2*-null spermatocytes and spermatids compared to WT controls (S1 Fig). Using spliceR, we analyzed the RNA-Seq data as described above and found no global upregulation of PTC-positive transcripts in either purified *Upf2*-null spermatocytes or in round spermatids (Fig 5C). Given that the canonical function of NMD is to degrade PTC-positive transcripts, these data do not support a role for UPF2-dependent NMD in scavenging a PTC-positive transcripts in germ cells. Instead, UPF2 appears to function to maintain the transcriptomic fidelity based on the large number of de-regulated transcripts upon *Upf2* ablation (Fig 5A). Together, these data, although unexpected, strongly suggest that the UPF2-mediated NMD pathway does not function to eliminate PTC-positive transcripts in germ cells, but is indeed required for maintaining transcriptomic fidelity during male germ cell development.

### Accumulation of alternatively spliced transcripts with longer 3’UTRs in *Upf2*-null spermatocytes and round spermatids

**Fig 6:**
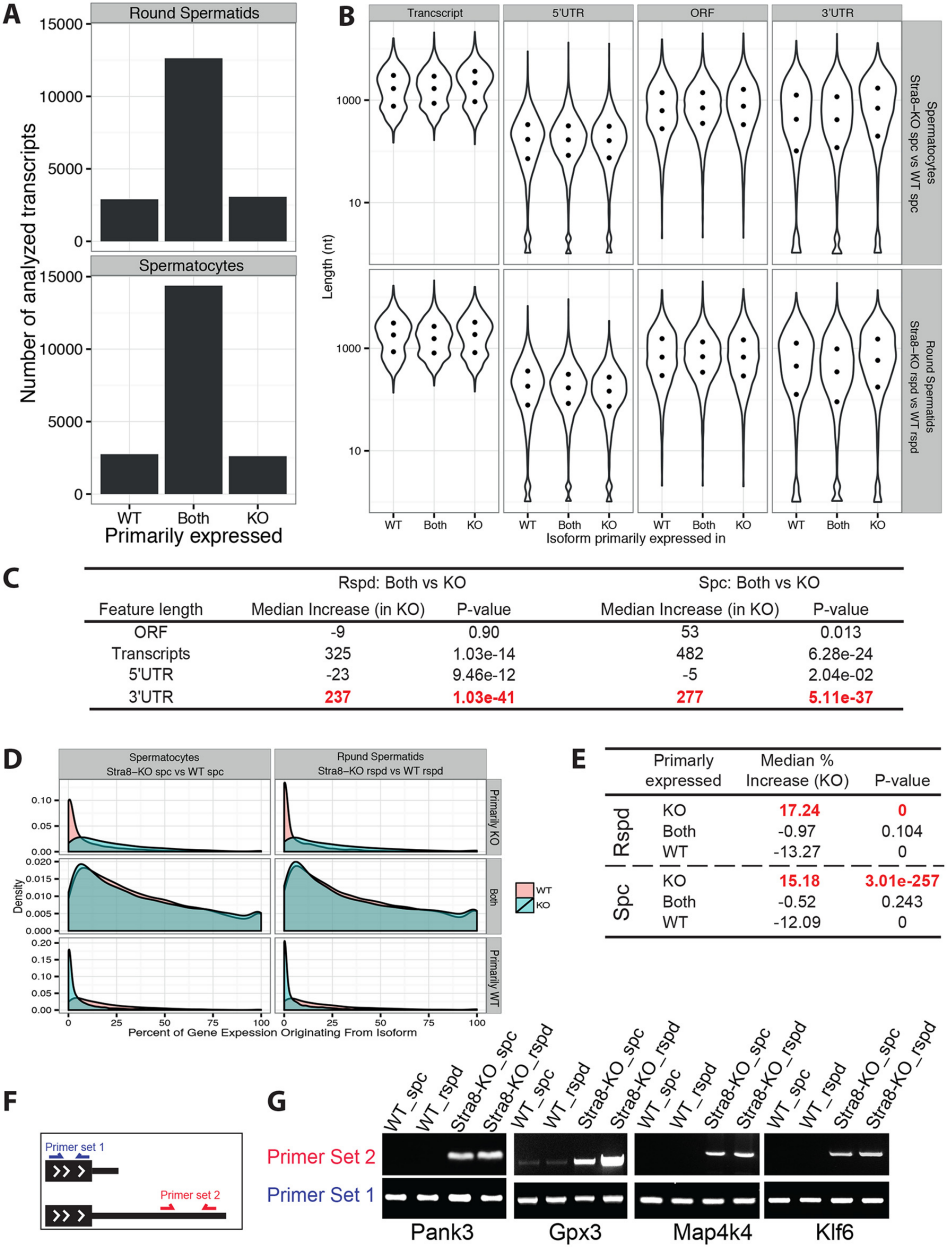
Longer 3’UTR, not PTC-containing transcripts, were accumulated in *Upf2*-null spermatocytes and round spermatids. (A) Bar plot showing the number of transcripts primarily expressed in WT, Stra8-KO or both genotypes, in spermatocytes (spc) and round spermatids (rspd). Cutoff: 1 normFPKM (see Methods and Materials). Classification of genes primarily expressed in WT, KO or both is given in S1 Table. (B) Violin plots showing the length distribution of the full-length transcripts, ORFs, 5’UTRs and 3’UTRs from the transcripts primarily expressed in WT, Stra8-KO or both genotypes. Rows indicate spermatocytes (spc) and round spermatids (rspd). The three dots in each violin plot indicate the 25^th^, 50^th^ (median) and 75^th^ percentile of the visualized data. Only transcripts derived from genes expressing multiple transcripts were analyzed. (C) A summary of statistics for the pairwise comparisons of the length distributions shown in B. (D) Distribution of the percentage by which each individual transcript contribute to their respective parent gene as measured in WT (red) and Stra8-KO (blue) in spermatocytes (left column) and round spermatids (right column). The analysis was performed for transcripts primarily expressed in WT, Stra8-KO or both genotypes (rows). Only transcripts derived from genes expressing multiple transcripts were analyzed. (E) A summary of statistics for the pairwise comparison of the length distributions shown in D. (F) Illustration of the strategy used for semi-quantitative PCR-based validation, with one pair from the protein-coding region (primer set 1) and the other from the 3’UTRs (primer set 2). (G) Semi-quantitative PCR-based validation of four randomly chosen transcripts with longer 3’UTRs (*Pank3*, *Gpx3*, *Map4k4* and *Klf6*). Note the alternative transcripts with longer 3’UTRs were readily detected in Stra8-KO samples but not in WT samples.

The lack of global PTC upregulation in *Upf2*-null germ cells contradicts the established canonical function of the NMD pathway in degrading aberrant PTC-containing transcripts [23, 24]. However, the severe spermatogenic disruptions in the absence of UPF2 clearly suggest that UPF2 plays an essential role independent of elimination of PTC-positive transcripts in male germ cells. Many defects, e.g., aberrant transcription, failure in exportation from the nucleus, incorrect splicing and/or alternatively polyadenylated, etc., all can cause the transcriptomic changes observed in *Upf2*-null testes and male germ cells. To gain mechanistic insight into spermatogenic disruption upon *Upf2* ablation, we performed further in-depth bioinformatics analyses by comparing the features of the full-length transcripts reconstructed from the RNA-Seq data. In the total testis, we found that the differentially upregulated transcripts (FDR < 0.05), derived from multi-isoform-expressing genes, displayed a median 3’UTR length of 1,446nt, which was significantly longer than both the non- (562 nt), or down-regulated (317 nt) transcripts (Fig 5D–5F). The differences in 3’UTR length were much greater than those in 5’UTR or ORF lengths (Fig 5D–5F), suggesting that the transcripts with longer 3’UTRs are selectively accumulated in the *Upf2*-deficient testes. As described earlier, the Stra8-KO testes contain much fewer spermatocytes and spermatids due to active depletion (Figs 3A and S4). Therefore, to further verify this finding, we performed similar analyses using RNA-Seq data from purified spermatocytes and round spermatids. Specifically, we discovered that >2,500 transcripts from multi-isoform-expressing genes with predicted ORFs were primarily expressed in Stra8-KO spermatocytes and round spermatids, suggesting a profound effect on gene expression upon *Upf2* inactivation (Fig 6A, S1 Table). Here, “primarily expressed” transcripts are defined as those expressed above 1 normFPKM in one genotype and below 1 normFPKM in the other genotype (see Methods and Materials). Consistent with our total testis analyses, the transcripts primarily expressed in purified *Upf2*-null spermatocytes and round spermatids also displayed significantly longer 3’UTRs compared to those expressed in control cells (mean difference >220 nt, p-value < 5.11E-37, Wilcoxon rank test) (Fig 6B and 6C).

To exclude the possibility that upregulation of transcripts with longer 3´UTRs merely reflects a general increase in expression of their parent genes, we further analyzed the fraction by which these transcripts contributed to the expression of their corresponding parent genes. Strikingly, in both total testis and purified spermatocytes and spermatids samples, the percentages by which the upregulated isoforms contributed to the expression of their parent genes were upregulated in the *Upf2-*deficient conditions (mean percentage increase >9.6%, p-value < 5.46e-10), thereby confirming the selective upregulation of these transcripts (Figs 6D, 6E and S5). Moreover, analyses of changes in average weighted 3’UTR length (weighted by the relative contribution of each isoform to the expression of its parent gene) further support this conclusion. Specifically, we find that genes containing isoforms with increased (> 20%) contribution to the expression of their parent genes have significantly longer average weighted 3’UTRs in both total and purified testis (median increase > 120nt, P < 4.86e-79, Mann-Whitney-U test) (S5 Fig). Similarly, but less pronounced, genes containing isoforms with decreased (> 20%) contribution to the expression of their parent have significantly shorter average weighted 3’UTRs in both total and purified testis (median decrease > 73nt, P < 8.18e-48, Mann-Whitney-U test) (S5 Fig). Finally, these findings could also be validated by semi-quantitative PCR analyses for selected genes (Fig 6F and 6G).

The combined bioinformatics analyses of the RNA-Seq datasets from both the total testis (Fig 5D–5F) and the purified spermatogenic cell types (Fig 6B–6G), clearly demonstrate that a group of mRNAs with significantly longer 3’UTRs accumulates in the absence of UPF2. Furthermore we note that this effect is most pronounced for midrange-expressed genes (5–50 FPKM/normFPKM) (S5 Fig), indicating that the effect is not caused by induction of transcription of genes/transcripts with relatively long 3'UTRs, but rather regulation of the relative stability of transcripts with longer 3’UTR’s. This finding is consistent with the data in several recent reports, in which *in vitro* reporter and cross-link immunoprecipitation (CLIP) assays demonstrated that UPF1, another core NMD factor, can bind the 3’UTRs, and selectively cause degradation of the mRNA transcripts with longer 3’UTRs *via* the NMD pathway [32–35]. Taken together, these data suggest that UPF2 can selectively eliminate alternative transcripts with longer 3’UTRs, which might contribute to a transcriptome enriched in transcripts with shorter 3’UTRs in late pachytene spermatocytes and round spermatids during spermatogenesis.

### Selective degradation of longer 3’UTRs derived from ubiquitously expressed genes by the NMD pathway contributes to the repertoire of shorter 3’UTR transcripts in spermatogenic cells

**Fig 7:**
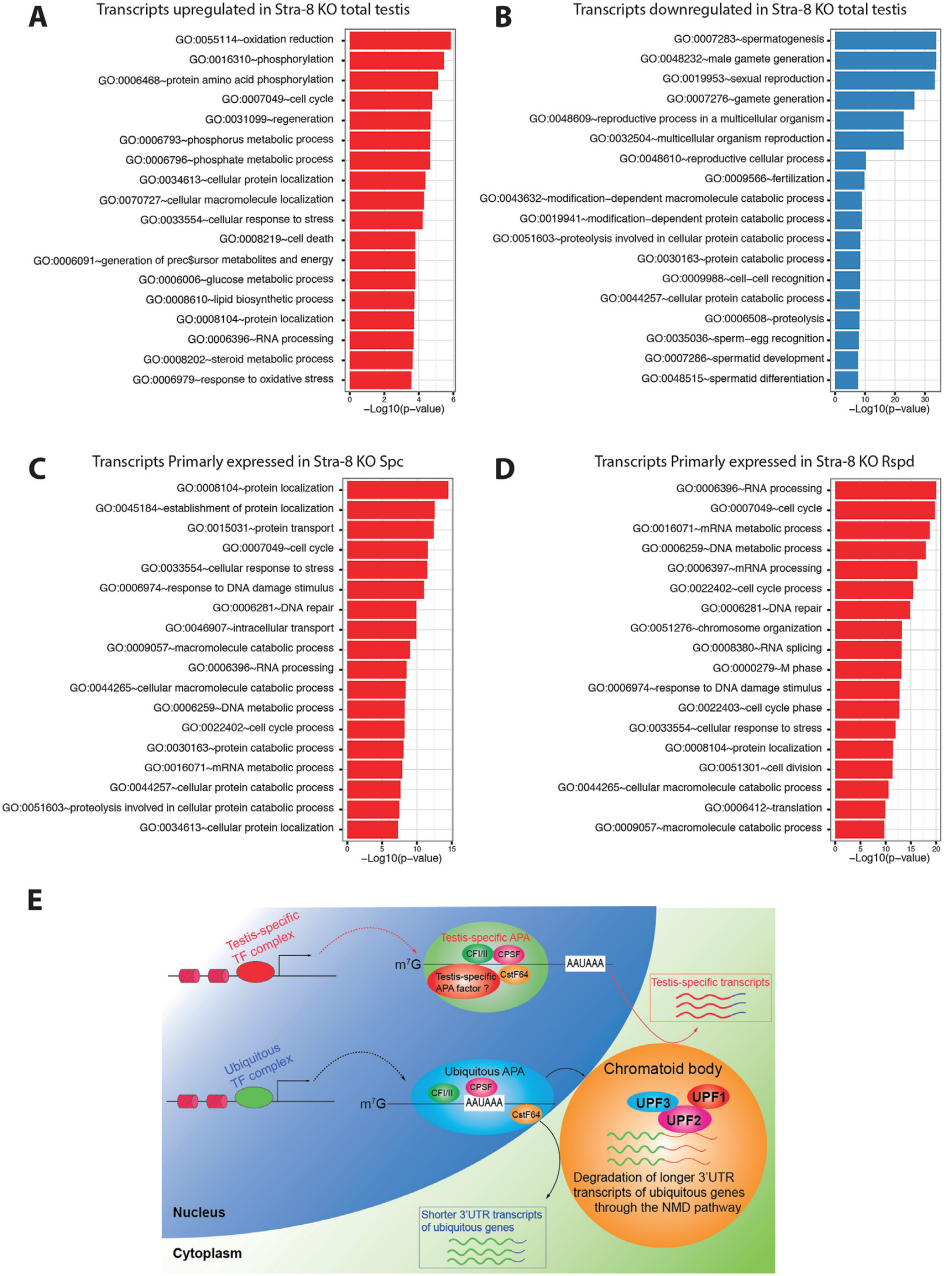
UPF2 selectively eliminates longer 3’UTR transcripts derived from ubiquitously expressed genes in spermatocytes and round spermatids. (A-B) Gene ontology (GO) analyses of differentially expressed genes (FDR < 0.05) for up- and down-regulated genes respectively (P<0.01; top 18 GO terms are shown). (C-D) Gene ontology (GO) analyses of genes with transcripts selectively upregulated upon *Upf2* inactivation in spermatocytes (Spc) and round spermatids (Rspd) respectively (P<0.01; top 18 GO terms are shown). (E) A working model for UPF2-mediated 3’UTR length control in male germ cells. While the testis-specific transcription factor (TF) complex containing the yet-to-be-identified testis-specific APA factors produces testis-specific gene transcripts with shorter 3’UTRs, the ubiquitous TF complex cooperates with the ubiquitous APA complex to generate both shorter and longer 3’UTR transcripts from ubiquitously expressed genes in male germ cells. The transcripts with longer 3’UTRs are then selectively degraded by the UPF2-directed NMD in the chromatoid body, leading to enrichment of shorter 3’UTR transcripts in haploid male germ cells.

It has been well documented that the testis is enriched in transcripts with shorter 3’UTRs, and this transcriptomic feature is essential for spermatogenesis and male fertility [6–9, 14]. At the transcriptional level, the germ cell-specific APA machinery, including testis-specific CstF64, is believed to specifically drives the production of shorter 3’UTR transcripts for numerous, well-known testis-specific genes (e.g., *Tnp1*, *Tnp2*, *Prm1* and *Prm2*) [5, 8, 9]. Although ubiquitously expressed, somatic genes can generate multiple transcripts with variable 3’UTR lengths in the testes, only the alternatively spliced transcripts with shorter 3’UTRs tend to be more stably expressed in the testis [5, 20, 51, 52], suggesting that the transcripts possessing longer 3’UTRs may have been eliminated through an as-yet-unknown mechanism. To test whether those accumulated transcripts with longer 3’UTRs in the absence of UPF2 are derived from ubiquitously expressed genes, we further conducted gene ontology (GO) analyses on de-regulated transcripts in both total testes and purified germ cell populations of Stra8-KO and control males. We discovered a significant enrichment in spermatogenesis-related genes among the downregulated transcripts (Fig 7A), which most likely resulted from decreased proportions of more advanced male germ cells (i.e. spermatocytes and spermatids) due to active depletion and/or disrupted testis-specific gene expression (Figs 4C and S4). In contrast, the upregulated transcripts in Stra8-KO testes (Fig 7B), or isoforms primarily expressed in *Upf2*-null germ cells (Fig 7C and 7D) were involved in a variety of biological processes that were not directly related to germ cell development. This suggests that longer 3’UTR transcripts derived from ubiquitously expressed genes selectively accumulated in *Upf2*-deficient germ cells. Detailed examination revealed that in Stra8-KO testes or purified *Upf2*-null germ cells, testis-specific genes, e.g., *Tnp1*, *Tnp2*, *Prm1*, and *Prm2*, expressed the same number of isoforms as those in WT controls, which is usually one or a few (S2 Table, highlighted in yellow), whereas ubiquitous genes produced many more isoforms, among which the ones with longer 3’UTRs were significantly up-regulated (Fig 6 and S2 Table). Taken together, these data support the production of testis-specific transcripts by germ cell-specific APA factors, and are also consistent with the notion that UPF2 selectively degrades longer 3’UTR transcripts derived from ubiquitously expressed genes in male germ cells during spermatogenesis. These two events may both contribute to the establishment of a repertoire of shorter 3’UTR transcripts in the developing male germ cells in the testis.

## Discussion

3’UTRs contain conserved binding sites for both miRNAs and RNA-binding proteins [10, 11, 16]. Transcripts with longer 3’UTRs tend to have more such binding sites, and thus, are subject to more comprehensive post-transcriptional regulation. In contrast, transcripts with shorter 3’UTRs could be more stable and more efficient in translation [10, 17]. Interestingly, it has been documented that more than half of the mammalian mRNA genes utilize the APA machinery to generate multiple transcripts with variable 3’UTR lengths, thereby altering their post-transcriptional fates, including mRNA stability, transportation and translational efficiency [7]. Increasing lines of evidence also suggest that the 3’UTR length control serves as a critical mechanism through which the cells and organs establish and maintain their transcriptome identity and functional status. For example, highly proliferative or cancerous cells tend to have a transcriptome enriched in transcript isoforms bearing shorter 3’UTRs, which is believed to enhance transcript stability and translational efficiency [16, 17]. In contrast, neuronal cells express abundant long 3’UTR transcripts, which allow for higher-order regulation by small RNAs and RNA-binding proteins [18, 19, 21, 22, 25]. Unlike the neuronal cells, developing male germ cells, especially spermatocytes and round spermatids, exhibit a transcriptome enriched in short 3’UTR transcripts, which is essential for normal male germ cell development and male fertility [6–9]. The necessity of 3’UTR length control for spermiogenesis (the haploid phase of spermatogenesis) is likely due to the fact that proteins required for late stages of sperm assembly (e.g. chromatin condensation/elongation, and flagellogenesis) mostly need to be translated using preexisting transcripts that are synthesized and stored in late pachytene spermatocytes and round spermatids, and these proteins need to be translated in a highly efficient manner to meet the tightly regulated timeline for sperm assembly [51, 53].

Our transcriptome-wide analyses reveal that while germ cell-specific genes constantly produce shorter 3’UTR transcripts in either WT or *Upf2*-null germ cells, a large number of longer 3’UTR isoform transcripts derived mainly from ubiquitously expressed genes are selectively accumulated in *Upf2*-null germ cells. This finding strongly suggests that UPF2-mediated degradation of longer 3’UTR transcripts derived from ubiquitously expressed genes, together with testis-specific gene-derived shorter 3’UTR transcripts, both contribute to the characteristic, shorter 3’UTR transcriptomic repertoire in murine testes.

In somatic cells, ablation of UPF2 causes an accumulation of PTC-containing transcripts [27, 31, 54]. However, in male germ cells, UPF2 ablation does not lead to an apparent accumulation of PTC-containing transcripts. Previous reports [55, 56] have suggested that the testicular PTC-containing transcripts, as byproducts of the highly active alternative splicing events in the developing male germ cells, must be eliminated efficiently. However, based on our data, this function must be mediated through a UPF2-independent NMD degradation pathway, which remains elusive and needs to be elucidated in the future. The novel role of UPF2 in eliminating longer 3’UTR transcripts derived from ubiquitously expressed genes in the male germ cells is different from its canonical NMD role in degrading PTC-containing transcripts. Consistent with our discovery, a recent study utilizing 3’UTR mRNA reporter coupled with high-throughput sequencing assays has demonstrated that decay of transcripts with longer 3´UTRs requires UPF2 in Hela cells [57]. Nevertheless, a key question remains: how does the UPF2-dependent NMD eliminate transcripts with longer 3’UTRs in the male germ cells? Several recent studies have demonstrated that UPF1 accumulates at 3’UTRs of full-length mRNA transcripts during the pioneer round of translation because UPF1 bound to other positions is gradually displaced by the termination ribosomes during translation [33–35, 58]. Consequently, transcripts with longer 3’UTRs tend to accommodate more UPF1-containing NMD complexes compared to the shorter 3’UTR transcripts, which can antagonize the stabilizing effects of poly(A)-binding proteins (e.g. PABPC1), leading to enhanced degradation of mRNA transcripts with longer 3’UTRs *via* the NMD pathway [32–35]. Because of the unavailability of a cell culture system for either meiotic or haploid male germ cells, one cannot directly recapitulate the above-discussed findings *in vitro*. However, numerous RNA-binding proteins, including PABPC1, PABPC2 and ELAVL1/HuR are known to be highly expressed in developing male germ cells, especially in spermatocytes and spermatids [59, 60], and they regulate mRNA stability and translational efficiency by binding the 3’UTRs [61–63]. Moreover, major NMD factors (e.g. UPF1, UPF2 and UPF3) all exhibit abundant expression in both meiotic and haploid germ cells (S1 Fig). Thus, it is conceivable that the UPF2-dependent NMD machinery can operate similarly to cause degradation of longer 3’UTR transcripts in developing male germ cells during spermatogenesis. Intriguingly, we observed that UPF2 is also required for spermatogonial development despite its relatively low expression levels. However, it is likely that UPF2 function through the canonical NMD pathway given that the characteristic shorter 3’UTR transcriptome has not yet been formed in spermatogonial populations.

Overall, our major findings include the following: *i)* UPF2 is specifically restricted to the RNA-processing center, the chromatoid body; *ii)* unlike in somatic cells, conditional ablation of *Upf2* does not upregulate PTC-positive transcripts in germ cells; *iii)* thousands of longer 3’UTR transcripts, were aberrantly accumulated in the *Upf2-*null spermatocytes and round spermatids. Based on these findings, we propose a working model for the UPF2-mediated NMD machinery in the 3’UTR length control in male germ cells. In this model, a yet-to-be-identified testis-specific APA machinery (as suggested in refs. [8, 9]) produces shorter 3’UTR transcripts from testis-specific genes, while the UPF2-mediated NMD machinery selectively eliminate transcript isoforms bearing longer 3’UTRs. These transcripts are mostly alternative isoforms of ubiquitously expressed genes and are decayed in the cytoplasmic RNA-processing center, the chromatoid body. The combined actions of these processes thereby shape the male germ cell-specific, shorter 3’UTR transcripts-enriched transcriptome in the testis (Fig 7E). These activities also support that CB is a critical RNA-processing center in haploid male germ cells, which is essential for spermatogenesis [37].

In summary, we have discovered that UPF2 is a new component of the chromatoid body, in which UPF2-mediated scavenging of longer 3’UTR transcripts derived from ubiquitously expressed genes is essential for spermatogenesis and male fertility. This mechanism may be utilized by other cell lineages as well in shaping cell/tissue-specific transcriptomic identity during development, adult physiology and pathophysiology.

## Materials and Methods

### Mouse breeding

Animal protocol for using mice (Protocol number 00494) was approved by Institutional Animal Care and Use Committee (IACUC) of the University of Nevada, Reno and are in accordance with the “Guide for the Care and Use of Experimental Animals” established by National Institutes of Health (NIH) (1996, revised 2011). The *Upf2* loxp mouse line was generated as described [31, 41]. The *Stra8-Cre* deletor line was purchased from the Jackson laboratory and backcrossed for 5 generations to the C57BL/6J background. Prospermatogonia-specific (Ddx4-KO) and spermatocytes/spermatids-specific (Stra8-KO) *Upf2* conditional knockout mice were generated by crossing *Upf2*^*fl/fl*^ mice with *Ddx4-Cre* and *Stra8-Cre* mice, respectively (S2 Fig). Genotyping was performed using tail PCR analyses as described [31, 41].

### Cell purification

Sertoli cells were purified using fluorescence-activated cell sorting (FACS) from transgenic mice (*Amh-Cre; mTmG*^*+/tg*^) in which membrane-tagged eGFP (mG) is specifically expressed in Sertoli cells. *Amh-Cre; mTmG*^*+/tg*^ mice were generated by crossing a Sertoli cell-specific Cre (*Amh-Cre*) line [64] with a dual fluorescence reporter line (*Rosa26-mTmG*^*tg/tg*^) [65]. Leydig cells were purified using FACS from *Cyp17-iCre; mTmG*^*+/tg*^ mice generated by crossing a Leydig cell-specific Cre (*Cyp17-iCre*) deletor line [66] with a dual fluorescence reporter line (*Rosa26-mTmG*^*tg/tg*^). The purities of both Sertoli and Leydig cells were >95% based on microscopic evaluation of the numbers of mG-positive vs. total cell. Spermatogonia were purified from P7 WT mouse testes, and spermatocytes and round spermatids were purified from adult WT and Stra8-KO mice testes using the STA-PUT method as described [67, 68]. The purities of spermatogonia, spermatocytes and spermatids were all >90% on the basis of microscopic examination and qPCR analyses of marker genes [67, 68].

### Histology

Hematoxylin-Eosin (HE) staining of paraffin sections of the testes was performed as described [69].

### RNA isolation, RT-PCR and qPCR

RNA was isolated using a RNA MiniPrep kit (Direct-zol, Zymo, Cat#R2050) following the manufacturer’s protocol. All RNA samples were treated by DNase I (Ambion, DNA-free Kit, Cat#AM1906) before reverse transcription and semi-quantitative or real-time quantitative PCR (qPCR) as described [69]. Sequences of PCR primers used are listed in S3 Table.

### Antibodies

The following antibodies were used in this study: anti-hUPF2 (Rabbit, a kind gift from Dr. Jens Lykke-Andersen. IF: 1:500 dilution; WB: 1:1000 dilution) [70], anti-MAEL (Guinea pig, a kind gift from Dr. Sadaki Yokota. IF: 1:200 dilution) [39], anti-DDX25 (Rat, a kind gift from Dr. Sadaki Yokota. IF: 1:500 dilution) [39], anti-SOHLH1 (Rabbit, a kind gift from Dr. Aleksandar Rajkovic. IF: 1:200 dilution) [71], anti-GCNA (Rat, a kind gift from Dr. George Enders. IF: 1:10 dilution) [44], anti-active CASPASE 3 antibody (Rabbit, Abcam, ab13847. IF: 1:500 dilution), anti-hUPF2/RENT2 antibody (Rabbit, Abcam, ab153830. IF: 1:500 dilution), and anti-WT1 (Rabbit, Santa Cruz, sc-192. IF: 1:50 dilution).

### Western blot and immunofluorescent staining

Western blot analyses were conducted as described previously [72]. Immunofluorescent staining of testicular cryosections was performed as described [73].

### RNA-Seq

Total RNA was isolated using the Trizol reagent (Invitrogen; Cat#15596–018) from whole WT and Stra8-KO (*Stra8-Cre;Upf2*^*fl/Δ*^) testes at the age of 6 weeks in biological triplicates, followed by DNase I treatment and an additional purification using the RNeasy Mini Kit (Qiagen, Cat#74104). RNA integrity and quantity were determined using the Agilent 2100 Bioanalyzer. Total RNA (2μg) was used to generate sequencing libraries using the TruSeq RNA sample prep kit-v2 (Illumina, Cat#15027387) according to the manufacturer’s instructions, with a size selection between 350bp and 500bp and a PCR cycle number at 10. Barcoded libraries were pooled and sequenced using an Illumina HiSeq2000 sequencer (100bp paired-end reads). A summary of sequence reads from the RNA-Seq analyses was listed in S4 Table.

Total RNA was isolated from spermatocytes and round spermatids purified from a pool of 8 WT and 12 Stra8-KO (*Stra8-Cre;Upf2*^*fl/Δ*^) testes in duplicates at the age of 6 weeks using a Direct-zol RNA MiniPrep kit (Zymo, # R2050) with on-column DNase I treatment. RNA quality and quantity were assessed using the Agilent 2100 Bioanalyzer. Total RNA (1.5μg) was used to prepare the RNA-Seq libraries, which were then sequenced on an Illumina HiSeq2000 sequencer, as described above. A summary of sequence reads from the RNA-Seq analyses was listed in S5 Table.

### Bioinformatics analysis

Raw sequences were checked for quality using the FASTQC tool (http://www.bioinformatics.bbsrc.ac.uk/projects/fastqc/). Ends were trimmed with fastx_trimmer (purified cell populations: f = 10, l = 78; Total testis: wt: f = 11, Stra8-KO: f = 12) and then the fastq_quality_trimmer was used with parameter t = 30. The resulting trimmed sequences were mapped with Tophat v. 2.0.9 [74] (Default settings plus —b2-very-sensitive, -r 200 and—mate-std-dev to 100.) [74], using Ensembl NCBIM37 (Hg19) as reference transcriptome (provided through Illumnia’s iGenome). Mapped RNA-Seq data were assembled with Cufflinks v. 2.1.1 [49] (default settings plus—frag-bias-correct,—max-bundle-length 1e7, and—multi-read-correct.) [49] using Ensembl NCBIM37, as well as a mask GTF-file containing noncoding and other auxiliary RNA species (Ensembl NCBI37 rRNA, misc_RNA, scRNA_pseudogene, snoRNA, snRNA, miRNA, TR_C_gene, tRNA, and mitochondrial RNA). For the total testis data a FDR< 0.05 was required for calling differential expression between WT and KO for genes and transcripts. No differential expression analysis was made on the purified spermatocytes and spermatids RNA-seq data since replicates were not available. The resulting full length transcripts were annotated with coding potential and classes of alternative splicing using the Bioconductor package spliceR with default settings as described elsewhere [50]. Briefly, spliceR annotated transcripts with the most upstream compatible Ensemble coding sequence (CDS), translate the downstream open reading frame (ORF) and output transcript features, including positions and lengths of ORF, 5’ untranslated region (UTR), and 3’UTR lengths. To account for normalization problems in the RNA-Seq libraries of purified spermatocytes and spermatids, the isoform data was quantile normalized using the normalize.quantiles() function available in preprocessCore package (v. 1.26.1) of R (v. 3.1.0). Here we refer to the units of the resulting values as normFPKM. All transcripts belonging to the same genes were then summed to get the gene expression levels. The fraction of gene expression originating from a transcript was calculated as (transcript expression) / (gene expression). Genes having a FPKM/normFPKM below 1 in either WT or KO samples were filtered out to ensure reliability of the fractions calculated. Analyses of isoform fractions and length distributions were conducted using the subset of genes with 2 or more expressed isoforms (cutoff 1 FPKM/normFPKM). The average weighted 3’UTR length for a gene G, with expression *e*_*G*_, which have *n* isoforms, expressed at levels *e*_*1*_*…e*_*i…*_*e*_*n*_ and with corresponding 3’UTR lengths *l*_*1*_*…l*_*i…*_*l*_*n*,_ was calculated as follows:

$$\text{average weighted }3’\text{UTR length}=\frac{\sum_{i=1}^{n}l_{i}*\frac{e_{i}}{e_{G}}}{n}$$

Where *e*_*i*_/*e*_*G*_ corresponds to the fraction of gene expression originating from transcript *i*.

Statistical analyses were performed using statistical software R v. 3.0.1., as indicated in figures/legends. Gene Ontology (GO) enrichment analysis was performed using DAVID (v6.7) online programs [75] with the default settings.

### Accession number

Data sets have been submitted to gene expression omnibus (GEO) under the accession number GSE55180.

## Supporting Information

S1 Fig Upf2 is ubiquitously expressed in multiple organs with the highest expression levels in spermatocytes and round spermatids in murine testes. (A) Western blot analyses of UPF2 protein levels in multiple organs in mice. ACTIN was used as a loading control. ES, Embryonic stem cells. (B) qPCR analyses of *Upf2* mRNA levels in developing testes in mice (E, Embryonic day; P, Postnatal day). Data were presented as means±SD, n = 3. (C) qPCR analyses of multiple nonsense-mediated decay (NMD) factors (*Upf1*, *Upf2*, *Upf3a*, *Upf3b*, *Smg1*, *Smg5*, *Smg6* and *Smg7*) in purified testicular cell populations including spermatogonia (spg), spermatocytes (spc), round spermatids (rspd), Sertoli cells (Sertoli) and Leydig cells (Leydig). (D) qPCR analyses of *Upf2* mRNA levels in spermatocytes (spc) and round spermatids (rspd) purified from WT and Stra8-KO testes.
(PDF)

S2 Fig Generation of male germline-specific *Upf2* conditional knockout mice. (A) A schematic diagram showing the critical events and timeline of male germ cell development in murine testes. After the completion of genome-wide de-methylation at embryonic day 13.5 (E13.5), male germ cells become mitotically arrested prospermatogonia between E13.5 and postnatal day 3 (P3), followed by the first wave of spermatogenesis upon puberty. Ddx4-Cre and Stra8-Cre deletor lines express Cre mRNA/protein in prospermatogonia as early as E15.5 and P3, respectively. However, the full penetrance of Cre-mediated recombination does not occur until P14 when the Stra8-Cre line is used. (B) Breeding strategy used for generating prospermatogonia-specific Upf2 knockout mice (Ddx4-Cre;*Upf2*^*fl/Δ*^ or Ddx4-KO). (C) Breeding strategy for generating spermatocytes and spermatids-specific *Upf2* knockout mice (*Stra8-Cre;Upf2*^*fl/Δ*^ or Stra8-KO).
(PDF)

S3 Fig Verification of the “Sertoli-cell-only syndrome” in Ddx4-KO testes at P10. Double immunofluorescent staining of WT1, a Sertoli cell marker, and GCNA, a germ cell marker, showed that only Sertoli cells are present in Ddx4-KO testes at postnatal day 10 (P10), resembling the “Sertoli-cell-only syndrome” in humans. Scale bar = 30μm.
(PDF)

S4 Fig Spermatogenic disruptions in developing and adult testes of Stra8-KO mice. Histology of WT and Stra8-KO testes at postnatal day 12 (P12), P14, P17, P21, P35 and 10 months is shown. Delayed entry into the meiotic phase is evident at P12 based on much fewer meiotic germ cells in Stra8-KO testes compared to WT testes. From P14 onwards, numerous vacuoles (*) are present in the seminiferous tubules of Stra8-KO testes, suggesting massive germ cell depletion. At the age of 10 months, while some tubules still contain various stages of spermatocytes (blue arrows) and spermatids (blue arrowheads), the majority of the tubules contain only Sertoli cells (red arrows) in Stra8-KO testes. Scale bar = 50μm.
(PDF)

S5 Fig Accumulation of longer 3’UTR, not PTC-containing transcripts, in *Upf2*-null testes, spermatocytes and round spermatids. (A) Distribution of the percentage by which individual transcripts contribute to their respective parent genes in total WT and *Upf2*-null (Upf2_KO) testes. Note that only transcripts derived from genes expressing multiple transcripts were analyzed. (B) Test and summary statistics for the pairwise comparison of the length distributions shown in A. (C) For each multi-isoform gene expressed in both conditions (all isoforms > 1 FPKM in both conditions) in the total testis data, the average 3’UTR length, weighted by the relative contribution of each isoform to the gene expression, was calculated. For each gene the change in the average weighted 3’UTR length, between WT and Stra8-KO, was plotted (y-axis) as a function of the mean gene expression (x-axis). The genes are divided based on whether they contain an isoform with a change in its relative contribution to the expression of its parent gene (here defined as a change of minimum 20% between conditions) resulting in 4 subsets (columns): a) Genes without changing isoforms (n = 4,636), b) genes with isoform(s) with a increased contribution to the expression of its parent genes (n = 413), c) genes with isoform(s) with a decreased contribution to the expression of its parent genes (n = 674) and finally d) genes with isoforms displaying both increased and decreased to the expression of their parent genes (n = 402). (D) Same as in C, but done for each of the two purified cells RNA-Seq datasets (rows) using an expression cutoff of 1 normFPKM. The number of genes in each subset are as follows: round spermatides; a) n = 4135, b) n = 595, c) n = 566, d) n = 311and spermatocytes; a) n = 4571, b) n = 534, c) n = 390, d) n = 381. (E) Violin plots showing distributions of changes in the average weighted 3’UTR length for each of the 4 subsets of genes (calculations and subsets as described in C) in total testis. The three dots in each violin plot indicate the 25^th^, 50^th^ (median) and 75^th^ percentile of the visualized data. (F) Summary and test statistics (Mann-Whitney-U test) for the subsets shown in E, when compared to the none-changing “a” subset. (G) Violin plots showing distributions of changes in the average weighted 3’UTR length for each of the 4 subsets of genes (calculations and subsets as described in D), in each of the two purified cell populations. The three dots in each violin plot indicate the 25^th^, 50^th^ (median) and 75^th^ percentile of the visualized data. (H) Summary and test statistics (Mann-Whitney-U test) for the subsets shown in (G) when compared to the none-changing “a” subset.
(PDF)

S1 Table Transcripts primarily expressed in WT and Upf2-null spermatocytes (spc) and WT and Upf2-null round spermatids (rspd). (XLSX)

S2 Table Differentially expressed transcripts between WT and Stra8-KO whole testes determined through RNA-Seq. (XLSX)

S3 Table Primers used in this study. (XLSX)

S4 Table Number of reads from RNA-Seq analyses on WT and Stra8-KO total testes (n = 3). (DOCX)

S5 Table Number of reads obtained from RNA-Seq analyses on pooled spermatocytes (spc) and round spermatids (rspd) purified from WT and Stra8-KO teste. (DOCX)
