# Supplementary figures and images for: UPF2-Dependent Nonsense-Mediated mRNA Decay Pathway Is Essential for Spermatogenesis by Selectively Eliminating Longer 3'UTR Transcripts
Source: PLoS Genet. 2016 May 5;12(5):e1005863. doi: 10.1371/journal.pgen.1005863 (PMC4858225; doi:10.1371/journal.pgen.1005863)

**A**

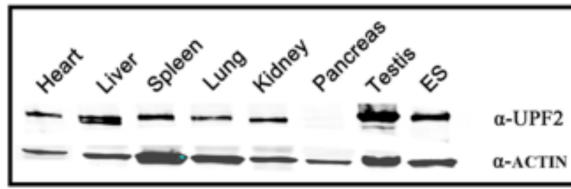

**B**

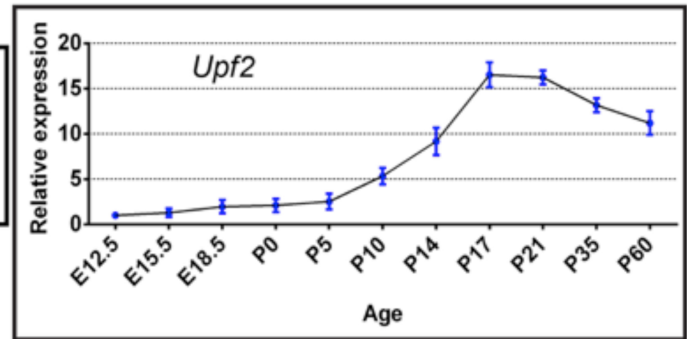

**C**

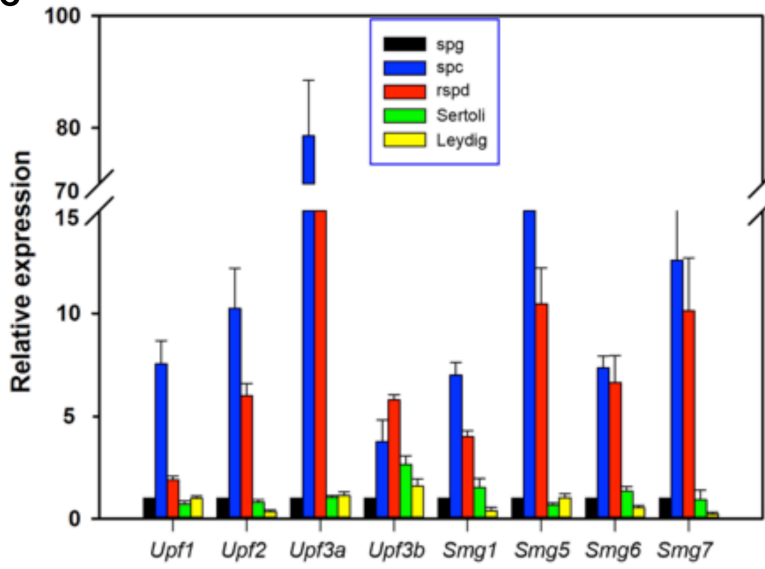

**D**

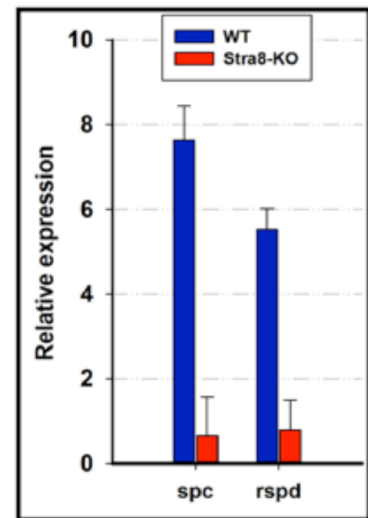

Supplement: S1 Fig — (A) Western blot analyses of UPF2 protein levels in multiple organs in mice. ACTIN was used as a loading control. ES, Embryonic stem cells. (B) qPCR analyses of Upf2 mRNA levels in developing testes in mice (E, Embryonic day; P, Postnatal day). Data were presented as means±SD, n = 3. (C) qPCR analyses of multiple nonsense-mediated decay (NMD) factors (Upf1, Upf2, Upf3a, Upf3b, Smg1, Smg5, Smg6 and Smg7) in purified testicular cell populations including spermatogonia (spg), spermatocytes (spc), round spermatids (rspd), Sertoli cells (Sertoli) and Leydig cells (Leydig). (D) qPCR analyses of Upf2 mRNA levels in spermatocytes (spc) and round spermatids (rspd) purified from WT and Stra8-KO testes. (PDF) [file pgen.1005863.s001.pdf]

A

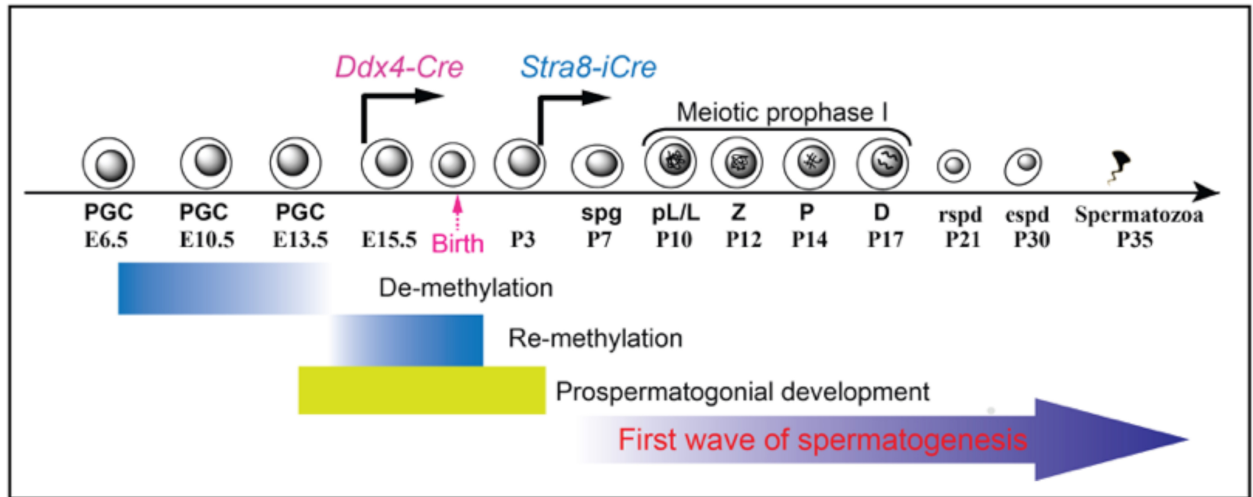

B

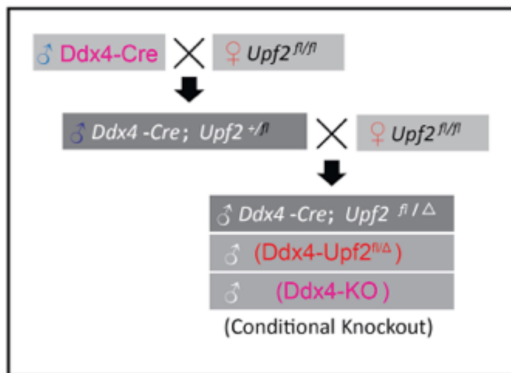

C

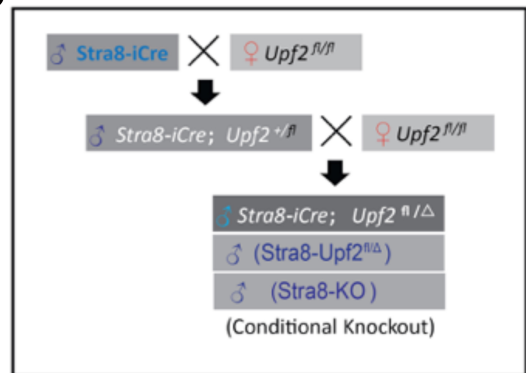

Supplement: S2 Fig — (A) A schematic diagram showing the critical events and timeline of male germ cell development in murine testes. After the completion of genome-wide de-methylation at embryonic day 13.5 (E13.5), male germ cells become mitotically arrested prospermatogonia between E13.5 and postnatal day 3 (P3), followed by the first wave of spermatogenesis upon puberty. Ddx4-Cre and Stra8-Cre deletor lines express Cre mRNA/protein in prospermatogonia as early as E15.5 and P3, respectively. However, the full penetrance of Cre-mediated recombination does not occur until P14 when the Stra8-Cre line is used. (B) Breeding strategy used for generating prospermatogonia-specific Upf2 knockout mice (Ddx4-Cre;Upf2fl/Δ or Ddx4-KO). (C) Breeding strategy for generating spermatocytes and spermatids-specific Upf2 knockout mice (Stra8-Cre;Upf2fl/Δ or Stra8-KO). (PDF) [file pgen.1005863.s002.pdf]

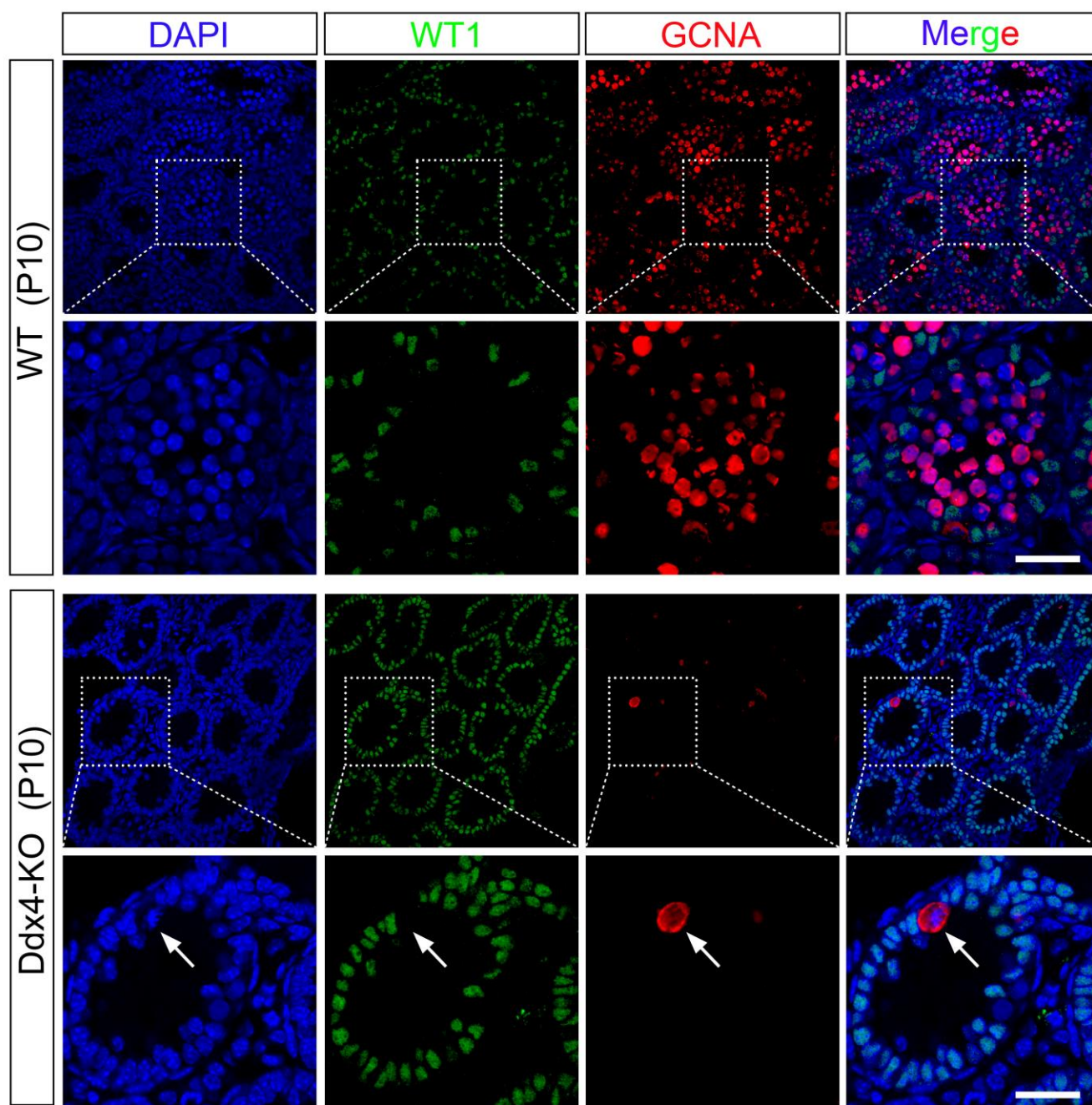

Supplement: S3 Fig — Double immunofluorescent staining of WT1, a Sertoli cell marker, and GCNA, a germ cell marker, showed that only Sertoli cells are present in Ddx4-KO testes at postnatal day 10 (P10), resembling the “Sertoli-cell-only syndrome” in humans. Scale bar = 30μm. (PDF) [file pgen.1005863.s003.pdf]

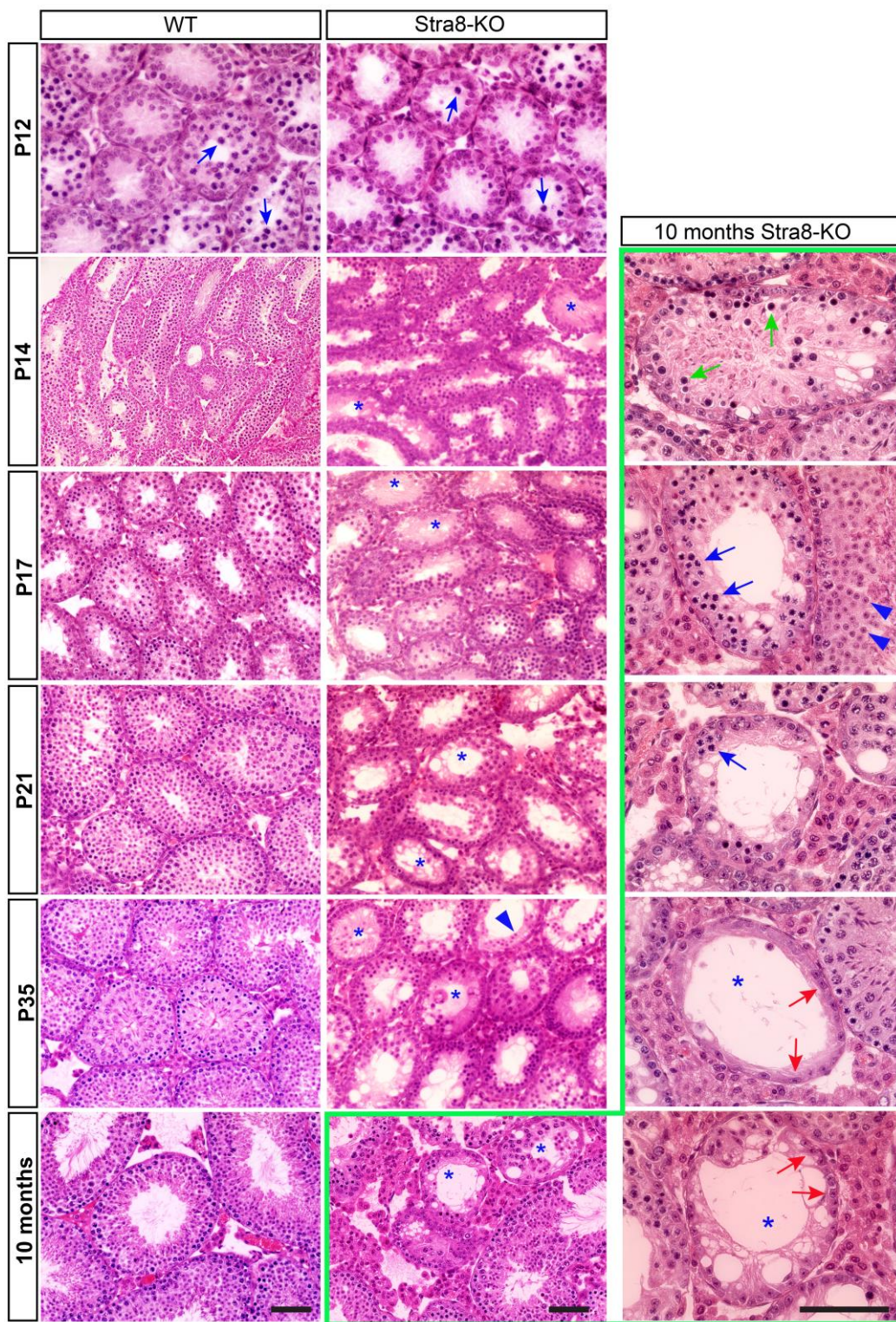

Supplement: S4 Fig — Histology of WT and Stra8-KO testes at postnatal day 12 (P12), P14, P17, P21, P35 and 10 months is shown. Delayed entry into the meiotic phase is evident at P12 based on much fewer meiotic germ cells in Stra8-KO testes compared to WT testes. From P14 onwards, numerous vacuoles (*) are present in the seminiferous tubules of Stra8-KO testes, suggesting massive germ cell depletion. At the age of 10 months, while some tubules still contain various stages of spermatocytes (blue arrows) and spermatids (blue arrowheads), the majority of the tubules contain only Sertoli cells (red arrows) in Stra8-KO testes. Scale bar = 50μm. (PDF) [file pgen.1005863.s004.pdf]
