# Supplementary material for: UPF2-Dependent Nonsense-Mediated mRNA Decay Pathway Is Essential for Spermatogenesis by Selectively Eliminating Longer 3'UTR Transcripts
Source: PLoS Genet. 2016 May 5;12(5):e1005863. doi: 10.1371/journal.pgen.1005863 (PMC4858225; doi:10.1371/journal.pgen.1005863)
Supplement: S5 Fig — (A) Distribution of the percentage by which individual transcripts contribute to their respective parent genes in total WT and Upf2-null (Upf2_KO) testes. Note that only transcripts derived from genes expressing multiple transcripts were analyzed. (B) Test and summary statistics for the pairwise comparison of the length distributions shown in A. (C) For each multi-isoform gene expressed in both conditions (all isoforms > 1 FPKM in both conditions) in the total testis data, the average 3’UTR length, weighted by the relative contribution of each isoform to the gene expression, was calculated. For each gene the change in the average weighted 3’UTR length, between WT and Stra8-KO, was plotted (y-axis) as a function of the mean gene expression (x-axis). The genes are divided based on whether they contain an isoform with a change in its relative contribution to the expression of its parent gene (here defined as a change of minimum 20% between conditions) resulting in 4 subsets (columns): a) Genes without changing isoforms (n = 4,636), b) genes with isoform(s) with a increased contribution to the expression of its parent genes (n = 413), c) genes with isoform(s) with a decreased contribution to the expression of its parent genes (n = 674) and finally d) genes with isoforms displaying both increased and decreased to the expression of their parent genes (n = 402). (D) Same as in C, but done for each of the two purified cells RNA-Seq datasets (rows) using an expression cutoff of 1 normFPKM. The number of genes in each subset are as follows: round spermatides; a) n = 4135, b) n = 595, c) n = 566, d) n = 311and spermatocytes; a) n = 4571, b) n = 534, c) n = 390, d) n = 381. (E) Violin plots showing distributions of changes in the average weighted 3’UTR length for each of the 4 subsets of genes (calculations and subsets as described in C) in total testis. The three dots in each violin plot indicate the 25th, 50th (median) and 75th percentile of the visualized data. (F) Summary a [file pgen.1005863.s005.pdf]

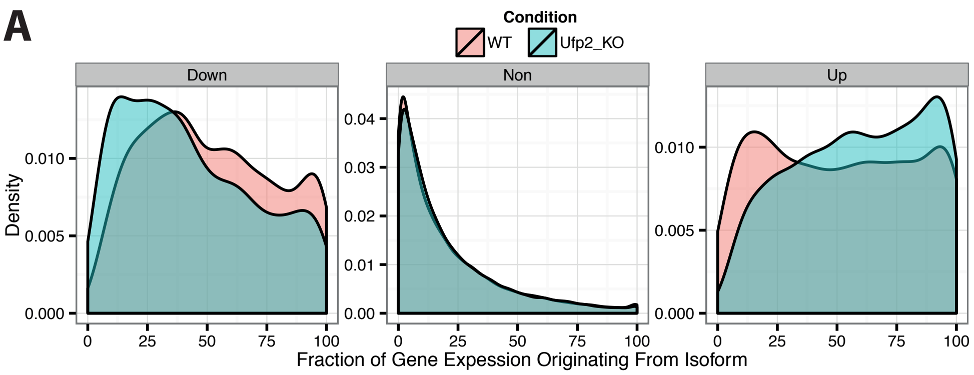

B

Differentially Regulated	Median % Increase (KO)	P-value
Up	**9.66**	**5.45e-10**
Non	0.37	4.05e-05
Down	-10.51	1.60e-26

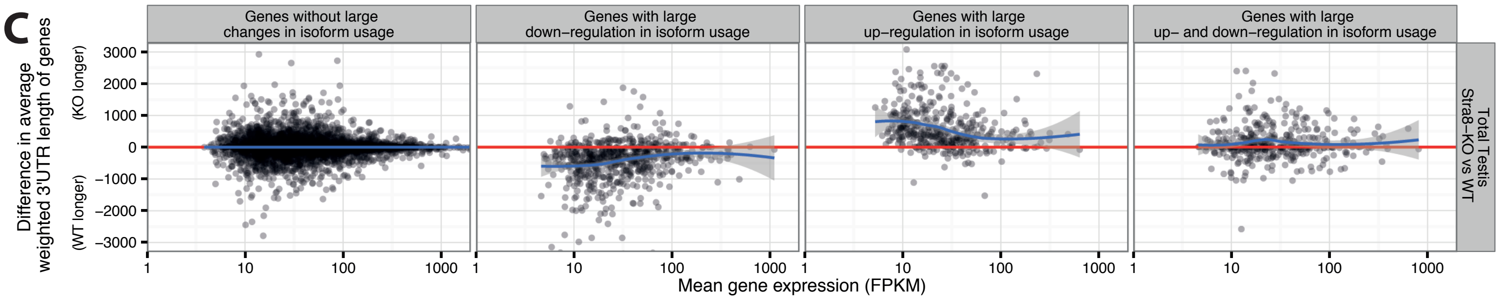

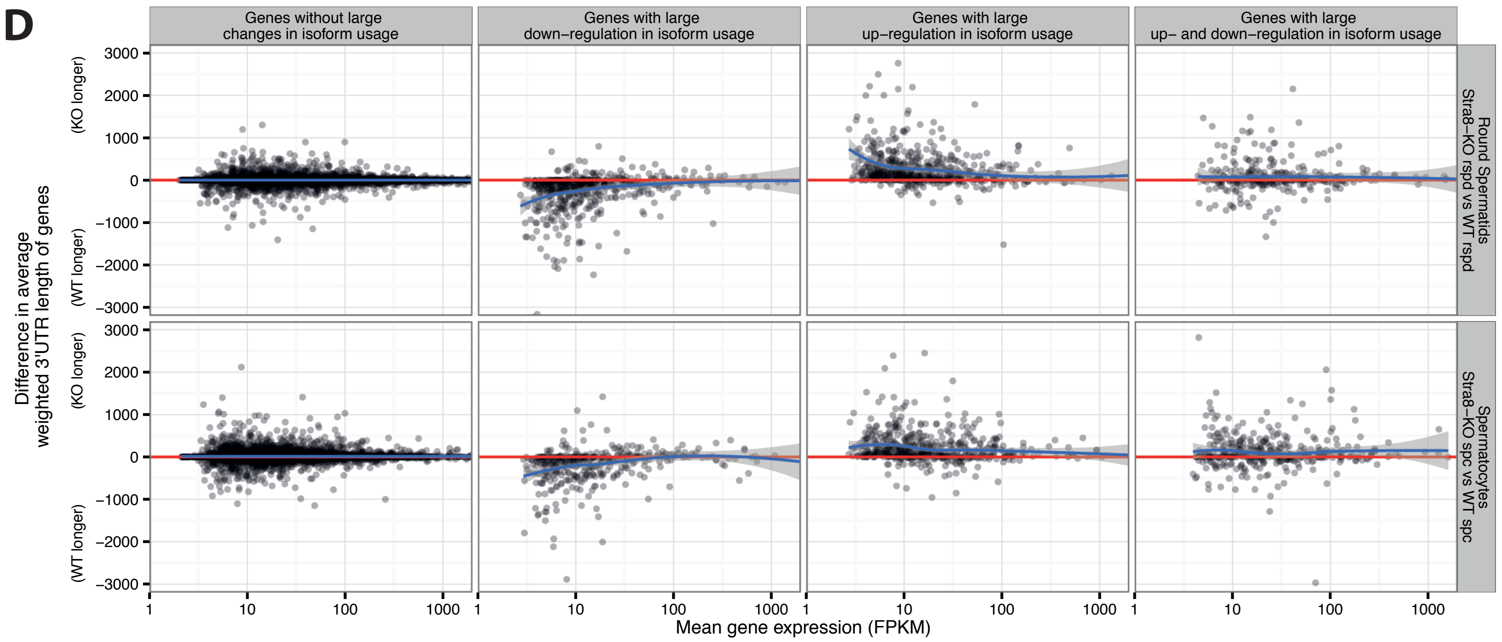

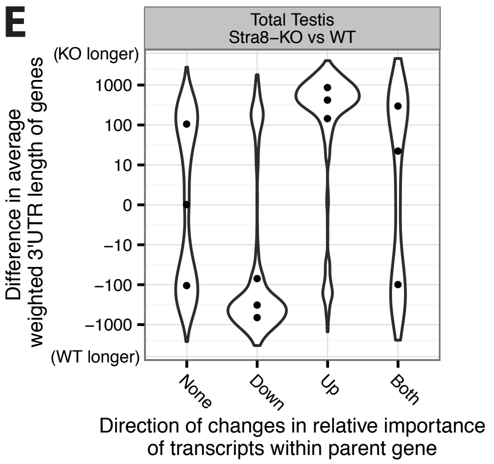

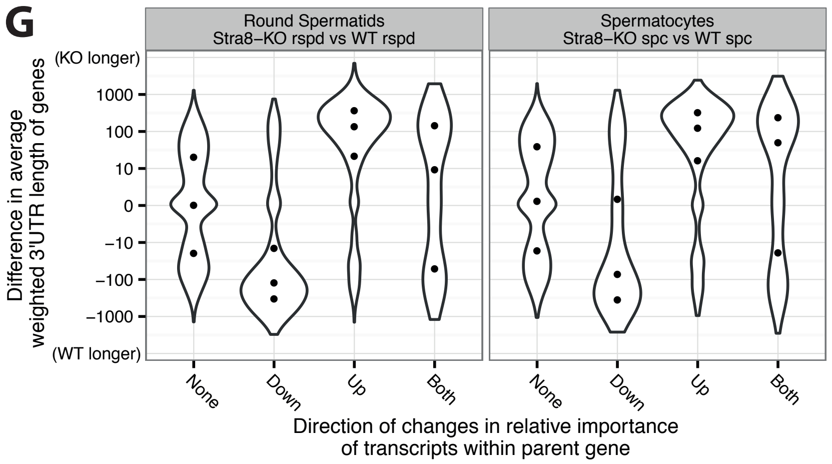

F

Relative importance	Median Avg 3'UTR Increase (KO) vs None	P-value
Down	-319.9 nt	1.79e-113
Up	423.6 nt	1.32e-104
Both	21.3 nt	8.42e-06

H

	Relative importance	Median Avg 3'UTR Increase (KO) vs None	P-value
Rrspd	Down	-123.5 nt	1.09e-106
	Up	133.6 nt	9.71e-125
	Both	8.2 nt	2.68e-05
Spc	Down	-73.7 nt	8.18e-48
	Up	120.3 nt	4.86e-79
	Both	48.6 nt	3.55e-12
