# Supplementary material for: UPF2-Dependent Nonsense-Mediated mRNA Decay Pathway Is Essential for Spermatogenesis by Selectively Eliminating Longer 3'UTR Transcripts
Source: PLoS Genet. 2016 May 5;12(5):e1005863. doi: 10.1371/journal.pgen.1005863 (PMC4858225; doi:10.1371/journal.pgen.1005863)
Supplement: S4 Table — (DOCX) [file pgen.1005863.s009.docx]

**S4 Table. Number of reads from RNA-Seq analyses on WT and Stra8-KO total testes (n=3).**

|  | | | |  |
| --- | --- | --- | --- | --- |
| **Total testis sample** | **Total pairs of raw reads** | **Processed pairs of reads** | **Mapped pairs of reads** | **Unique mapped pairs of reads** |
| WT1 | 26,497,004 | 25,667,860 | 24,033,020 | 23,746,561 |
| WT2 | 32,216,591 | 31,193,684 | 29,214,525 | 28,853,925 |
| WT3 | 30,230,076 | 29,283,717 | 27,439,042 | 27,084,938 |
| Stra8-KO_1 | 33,131,912 | 31,390,628 | 28,724,377 | 28,477,135 |
| Stra8-KO_2 | 33,515,147 | 31,773,343 | 29,197,004 | 28,941,047 |
| Stra8-KO_3 | 29,470,221 | 27,869,634 | 25,444,223 | 25,229,030 |
|  |  |  |  |  |
