# Supplementary material for: UPF2-Dependent Nonsense-Mediated mRNA Decay Pathway Is Essential for Spermatogenesis by Selectively Eliminating Longer 3'UTR Transcripts
Source: PLoS Genet. 2016 May 5;12(5):e1005863. doi: 10.1371/journal.pgen.1005863 (PMC4858225; doi:10.1371/journal.pgen.1005863)
Supplement: S5 Table — (DOCX) [file pgen.1005863.s010.docx]

| **S5 Table. Number of reads obtained from RNA-Seq analyses on pooled spermatocytes (spc) and round spermatids (rspd) purified from WT and Stra8-KO testes.** | | | | |
| --- | --- | --- | --- | --- |
| **Purified germ cell type** | **Total pairs of raw reads** | **Mapped pairs of reads** | **Unique mapped pairs of reads** |  |
| WT_spc | 21,078,391 | 17,125,266 | 13,103,538 |  |
| WT_rspd | 22,434,810 | 17,643,012 | 13,609,290 |  |
| Stra8-KO_spc | 25,936,402 | 24,191,239 | 18,850,182 |  |
| Stra8-KO_rspd | 24,747,828 | 21,200,353 | 13,224,061 |  |
